# TLR4 maintains Treg-mediated protection against adverse outcomes in a model of hepatic surgical stress

**DOI:** 10.1172/JCI194607

**Published:** 2026-03-02

**Authors:** Hongji Zhang, Yunwei Zhang, Tianxing Ren, Carolyn Tsung, Peng Song, Peng Xu, Guoliang Wang, Chunyan Cao, Changyan Wang, Ping Sun, Qi Zhang, Yanhong Zhu, Xin Zhong, Yong Guan, Xiaofei Zhang, Han Wang, Jinxiang Zhang, Hui Wang

**Affiliations:** 1 Department of Emergency Surgery, Union Hospital, and Department of Medical Genetics, School of Basic Medicine, Tongji Medical College, Huazhong University of Science and Technology, Wuhan, Hubei, China.; 2 Department of Surgery, University of Virginia, Charlottesville, Virginia, USA.; 3 Key Laboratory of Anesthesiology and Resuscitation, Huazhong University of Science and Technology, Ministry of Education, China.; 4 Department of Breast and Thyroid Surgery, People’s Hospital of Ningxia Hui Autonomous Region, Yinchuan, Ningxia, China.; 5 Department of Emergency Surgery, Union Hospital, Tongji Medical College, Huazhong University of Science and Technology, Wuhan, Hubei, China.; 6 Department of Hepatobiliary Surgery, Union Hospital, Tongji Medical College, Huazhong University of Science and Technology, Wuhan, Hubei, China.; 7 Department of Ultrasound, Union Hospital, Tongji Medical College, Huazhong University of Science and Technology, Wuhan, Hubei, China.; 8 Department of Medical Genetics, School of Basic Medicine, Tongji Medical College, Huazhong University of Science and Technology, Wuhan, Hubei, China.; 9 National “111” Center for Cellular Regulation and Molecular Pharmaceutics, Key Laboratory of Fermentation Engineering (Ministry of Education), Hubei University of Technology, Wuhan, Hubei, China.; 10 National Engineering Research Center for Nanomedicine, College of Life Science and Technology, Huazhong University of Science and Technology, Wuhan, Hubei, China.; 11 Center for Translational Medicine, Union Hospital, Tongji Medical College, Huazhong University of Science and Technology, Wuhan, China.; 12 Department of Gastroenterology, Tongji Hospital, Tongji Medical College, Huazhong University of Science and Technology, Wuhan, Hubei 430030, China.

**Keywords:** Hepatology, Immunology, Inflammation, Hypoxia, Surgery, Tregs

## Abstract

Surgical stress, such as hepatic ischemia-reperfusion (I/R) injury, induces excessive inflammation and adversely affects liver surgery outcomes. Regulatory T cells (Tregs) are crucial for immune homeostasis, yet their protective mechanisms against liver I/R injury remain unclear. In this study, we demonstrated that decreased hepatic Treg abundance correlates with increased liver injury in patients undergoing hepatic hemangioma resections. In murine models, Treg depletion worsened liver I/R injury. Bulk RNA-seq of hepatic Tregs showed enrichment of Toll-like receptor (TLR) signaling pathways, with flow cytometry identifying TLR4 as the most increased TLR after I/R. Treg-specific *Tlr4* knockout mice (Treg-*Tlr4*^–/–^ mice) exhibited exacerbated liver injury following I/R. Adoptive transfer of WT Tregs, but not *Tlr4*-deficient Tregs, alleviated liver injury in both Treg-depleted and Treg-*Tlr4*^–/–^ mice. Transcriptomic analysis revealed that IL-10 production was impaired in *Tlr4*-deficient Tregs. Mechanistically, *Tlr4*-deficient Tregs showed reduced activation of the MyD88/ERK/CREB pathway, resulting in diminished IL-10 production. *Myd88*^–/–^ and *IL-10*^–/–^ Tregs failed to confer protection against liver I/R injury, whereas exogenous IL-10 administration rescued the hepatic dysfunction in Treg-*Tlr4*^–/–^ mice. Our findings implicate the vital role of TLR4 in Tregs to mitigate liver I/R injury and offer a potential therapeutic option to reduce postoperative complications following liver surgery.

## Abstract 2

<strong>Toll-like Receptor 4 on Regulatory T Cell Alleviates Hepatic Surgical Stress by Promoting IL-10 Production through MyD88/ERK/CREB Signaling Pathway</strong>

## Graphical abstract

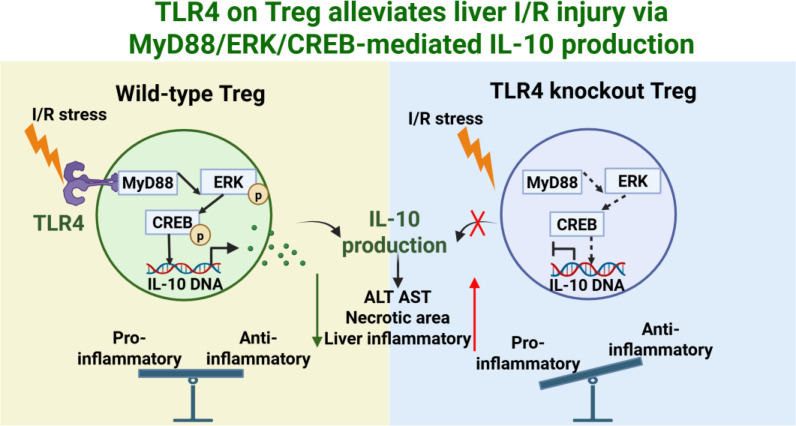

## Introduction

Major abdominal surgeries, including liver resection, induce surgical stress that disrupts immunologic and metabolic homeostasis, contributing to worsened postoperative outcomes and prolonged recovery (1, 2). A specific and common form of hepatic surgical stress is liver ischemia-reperfusion (I/R) injury, which often occurs during hepatic pedicle clamping — a temporary inflow occlusion technique used to minimize intraoperative hemorrhage (3, 4). This maneuver elicits a robust hepatic inflammatory response driven by damage-associated molecular patterns (DAMPs) (5, 6). Uncontrolled inflammation following I/R injury can result in liver dysfunction, graft rejection, and even mortality (7, 8). Therefore, mitigating the hepatic inflammatory response is crucial for alleviating surgical stress–induced liver injury (9). Although interventions such as perioperative carbohydrate loading (10) and steroid administration (11) have shown some promise in reducing liver I/R injury, current strategies remain suboptimal in preventing or reversing the immunopathological consequences. A clearer understanding of the immunoregulatory mechanisms underlying liver I/R injury is urgently needed.

Liver I/R injury initiates a complex cascade involving innate and adaptive immune responses, disrupting the balance between pro- and antiinflammatory mediators, ultimately exacerbating liver injury (3, 12, 13). Among the adaptive immune components, CD4^+^ T cells play a pivotal role in modulating liver I/R injury in both preclinical and clinical models (14). Regulatory T cells (Tregs), a specialized subset of CD4^+^ T cells marked by expression of the transcription factor Foxp3, suppress immune responses via direct cell contact and the secretion of immunomodulatory cytokines, such as IL-10 and TGF-β (15, 16). In contrast, T helper 17 cells (Th17 cells), a proinflammatory CD4^+^ T cell subtype, have been shown to exacerbate liver I/R injury (17). Recent studies suggest that restoring the Th17/Treg balance can mitigate I/R induced hepatic injury (17–19). Although the protective role of Tregs in mitigating IRI is established in other organs, including the kidney and brain (20, 21), the direct evidence demonstrating the role of Tregs in liver I/R injury remains limited, and the molecular mechanisms governing their immunosuppressive function are not fully defined.

Toll-like receptor 4 (TLR4), a major pattern recognition receptor for DAMPs, is a key driver of the inflammatory response during liver I/R injury (22). TLR4 activation in hepatocytes and Kupffer cells (KCs) has been shown to amplify local inflammatory signaling and exacerbates hepatic I/R injury (23). Interestingly, beyond its established proinflammatory functions in KCs and hepatocytes, TLR4 has also been shown to reduce Foxp3 expression in Tregs, impairing suppressive function in acute lung injury (24). Complementing this, classical TLR4 activation by *E*. *coli* lipopolysaccharide (LPS) perturbs immune homeostasis by shifting the Th17/Treg balance via TLR4/NF-κB signaling, indicating that TLR4 engagement can directly influence Treg programs and Treg/Th17 equilibria (25). However, the functional consequences of TLR4 signaling within Tregs in liver I/R injury remain largely unexplored and poorly understood. Understanding whether and how TLR4 activation directly modulates Treg suppressive function and stability during hepatic I/R could reveal immunomodulatory pathways distinct from its known proinflammatory actions in other cell types, potentially offering therapeutic targets to mitigate liver damage.

In this study, we demonstrated that hepatic Tregs are reduced following hepatic pedicle occlusion and that their abundance negatively correlates with postoperative liver injury severity in both clinical hepatic hemangioma resections and murine liver I/R models. Treg depletion exacerbates liver I/R injury in mice, confirming their protective role. Furthermore, using mice with Treg-specific TLR4 deficiency, we showed that TLR4 modulates the transcriptional profile of Tregs, promoting an immunosuppressive phenotype. Loss of TLR4 in Tregs aggravated liver I/R injury by impairing the TLR4/MyD88/ERK/CREB signaling axis and reducing IL-10 production. These findings reveal a previously unrecognized role of TLR4 in maintaining Treg-mediated protection during liver I/R injury and suggest avenues for immunotherapeutic intervention in the perioperative setting.

## Results

### Hepatic Tregs protect against liver I/R injury.

**Figure 1:**
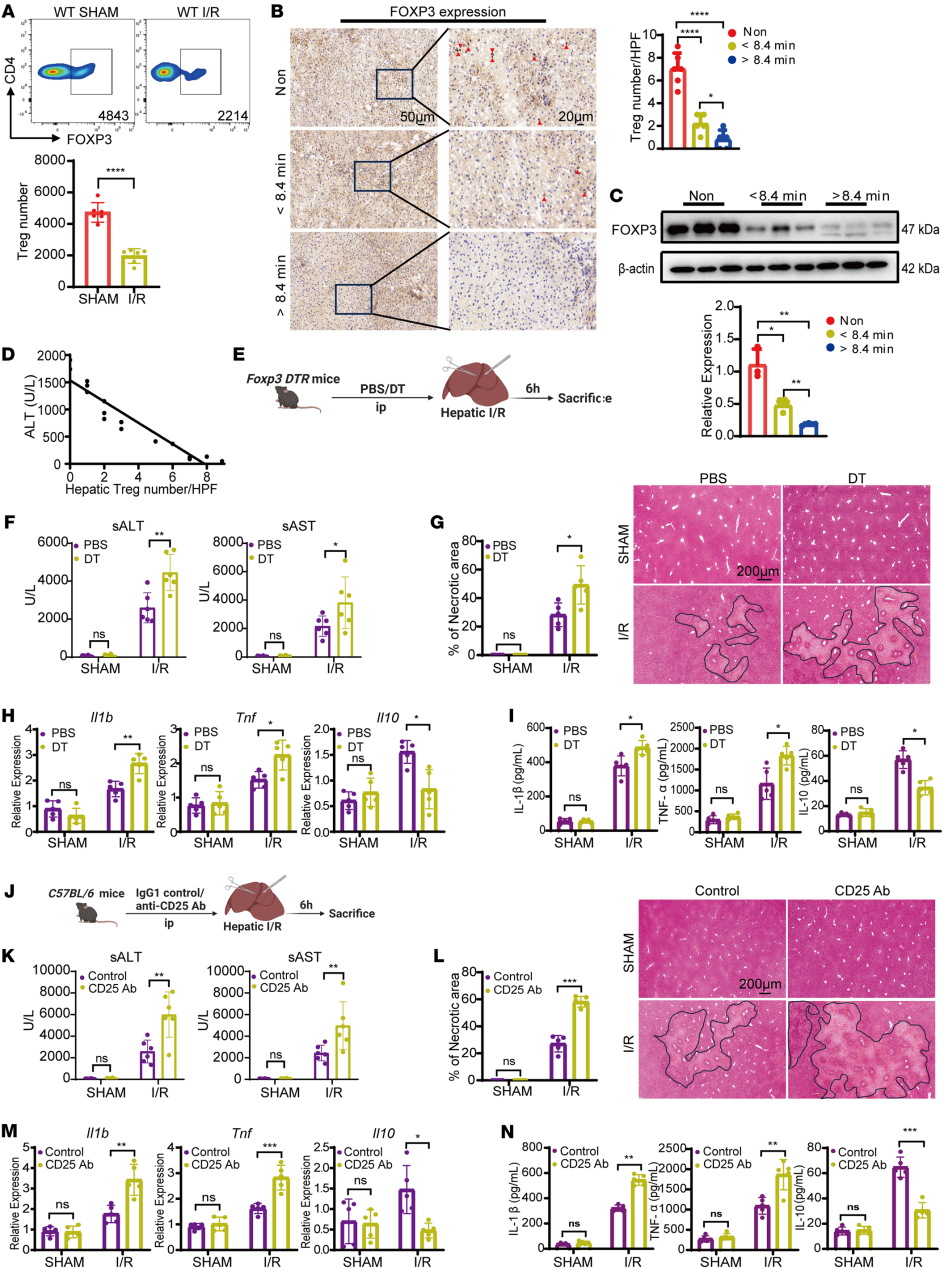
Hepatic Tregs protect against liver I/R injury. (**A**) The counts of hepatic CD4^+^FOXP3^+^ Tregs in WT mice before and after liver I/R (*n* = 6 per group). (**B**) IHC staining of FOXP3 in liver tissues of patients with hepatic hemangiomas who underwent partial hepatectomy, with or without hepatic pedicle blockage (*n* = 5–6 per group). Scale bars: 50 μm (left); 20 μm (right). (**C**) Western blot analysis of FOXP3 expression in the liver in these patients (*n* = 3 per group). (**D**) Correlation between these patients’ serum ALT and intrahepatic Tregs (*n* = 16, Spearman’s coefficient r = -0.983, *P* = 0.0089). (**E**) Experimental design for DT-induced Treg depletion in *Foxp3* DTR mice and its controls, followed by establishing the liver I/R model. (**F**) Serum ALT and AST levels in *Foxp3* DTR mice with or without Treg depletion before and after liver I/R (*n* = 6 per group). (**G**) Representative H&E staining images of ischemia liver from *Foxp3* DTR mice with or without Treg depletion before and after liver I/R (*n* = 5 per group); Scale bars: 200 μm. (**H**) Quantitative PCR analysis of *IL-1β*, *TNF-α*, and *IL-10* mRNA expression in liver from sham and I/R *Foxp3* DTR mice (*n* = 5 per group). (**I**) Serum IL-1β, TNF-α, and IL-10 levels from sham and I/R *Foxp3* DTR mice with or without Treg depletion (*n* = 5 per group). (**J**) Experimental design for anti-CD25 Ab-induced Treg depletion in WT mice and its controls, followed by establishing the liver I/R model. (**K**) Serum ALT and AST levels in WT mice with or without Treg depletion before and after liver I/R (*n* = 6 per group). (**L**) Representative H&E staining images of ischemia liver from WT mice with or without Treg depletion before and after liver I/R (*n* = 5 per group); Scale bars: 200 μm. (**M**) Quantitative PCR analysis of *Il1b*, *Tnf*, and *Il10* mRNA expression in liver from sham and I/R WT mice with or without Treg depletion (*n* = 5 per group). (**N**) Serum IL-1β, TNF-α, and IL-10 levels from sham and I/R WT mice with or without Treg depletion (*n* = 5 per group). Statistical analyses were performed using 1-way ANOVA (**A** and **B**), Spearman’s rank correlation coefficient (**C**), unpaired, 2-tailed *t* tests (**D**), and 2-way ANOVA with -Šidák’s post test (**F**–**I** and **K**–**N**). *P < 0.05, **P < 0.01, ***P < 0.001; ****P < 0.0001. ALT, Alanine aminotransferase; AST, Aspartate aminotransferase; DT, diphtheria toxin; Ab, Antibody.

Liver I/R injury triggers dramatic alterations in the hepatic immune cell landscape. We established a murine model of liver I/R and evaluated 10 hepatic immune cell subsets by flow cytometry. Compared with sham-operated controls, hepatic Tregs decreased significantly at 6 hours after I/R (Figure 1A), as did dendritic cells (DCs), B cells, and resident KCs. In contrast, neutrophils and monocytes increased markedly. No significant changes were observed in total CD4^+^ T cells, CD8^+^ T cells, NK cells, or NKT cells (Supplemental Figure 1, A–C; supplemental material available online with this article; https://doi.org/10.1172/JCI194607DS1).

To further assess the role of Tregs in liver I/R injury, we quantified hepatic Tregs in 16 hepatic hemangioma patients undergoing partial hepatectomy. Of these, 6 patients underwent surgery without intraoperative hepatic pedicle occlusion, while the remaining 10 underwent temporary pedicle clamping with a median occlusion time of 8.4 minutes. Pre- and postoperative characteristics are summarized in Supplemental Table 1. IHC and Western blot analyses revealed a greater reduction in Treg numbers, as evidenced by lower FOXP3 expression in liver tissue from patients with intraoperative occlusion compared with the nonischemic patients (Figure 1, B and C). Notably, patients with longer occlusion times (> 8.4 min) exhibited a more pronounced decrease in Treg numbers, as indicated by reduced FOXP3 levels compared with those with shorter occlusion times (< 8.4 min) (Figure 1, B and C). Furthermore, hepatic Treg numbers were inversely correlated with postoperative ALT levels (Figure 1D). These results suggest that hepatic Tregs are reduced following I/R and may play a protective role in limiting liver I/R injury.

To directly test the functional role of Tregs in liver I/R injury, we employed 2 Treg depletion strategies: DT administration in *Foxp3* DTR mice (26) and anti-CD25 antibody in WT mice (27) (Figure 1, E and J). In *Foxp3* DTR mice, DT-mediated Treg ablation significantly exacerbated liver injury 6 hours after reperfusion, as indicated by elevated serum ALT and AST levels compared with PBS-treated controls (Figure 1F). Histological analysis showed markedly larger necrotic areas in ischemia liver sections from DT-treated *Foxp3* DTR mice (Figure 1G). Moreover, Treg-depleted mice exhibited enhanced hepatic inflammation, characterized by increased expression of proinflammatory genes (*Il1b* and *Tnf*) and reduced levels of the antiinflammatory gene *Il10* in ischemic liver tissue (Figure 1H). The local inflammation was accompanied by an aggravated systemic inflammatory response, as indicated by increased serum levels of IL-1β, TNF-α, and decreased IL-10 (Figure 1I). Similarly, anti-CD25 antibody treatment in WT mice led to worsened hepatic injury following liver I/R, evidenced by elevated ALT and AST levels, extensive necrosis, and increased hepatic and systemic inflammatory responses (Figure 1, K–N). Together, these results demonstrate that hepatic Tregs mitigate liver I/R injury by limiting inflammatory responses and tissue damage.

### TLR4 is crucial for hepatic Treg function following liver I/R.

**Figure 2:**
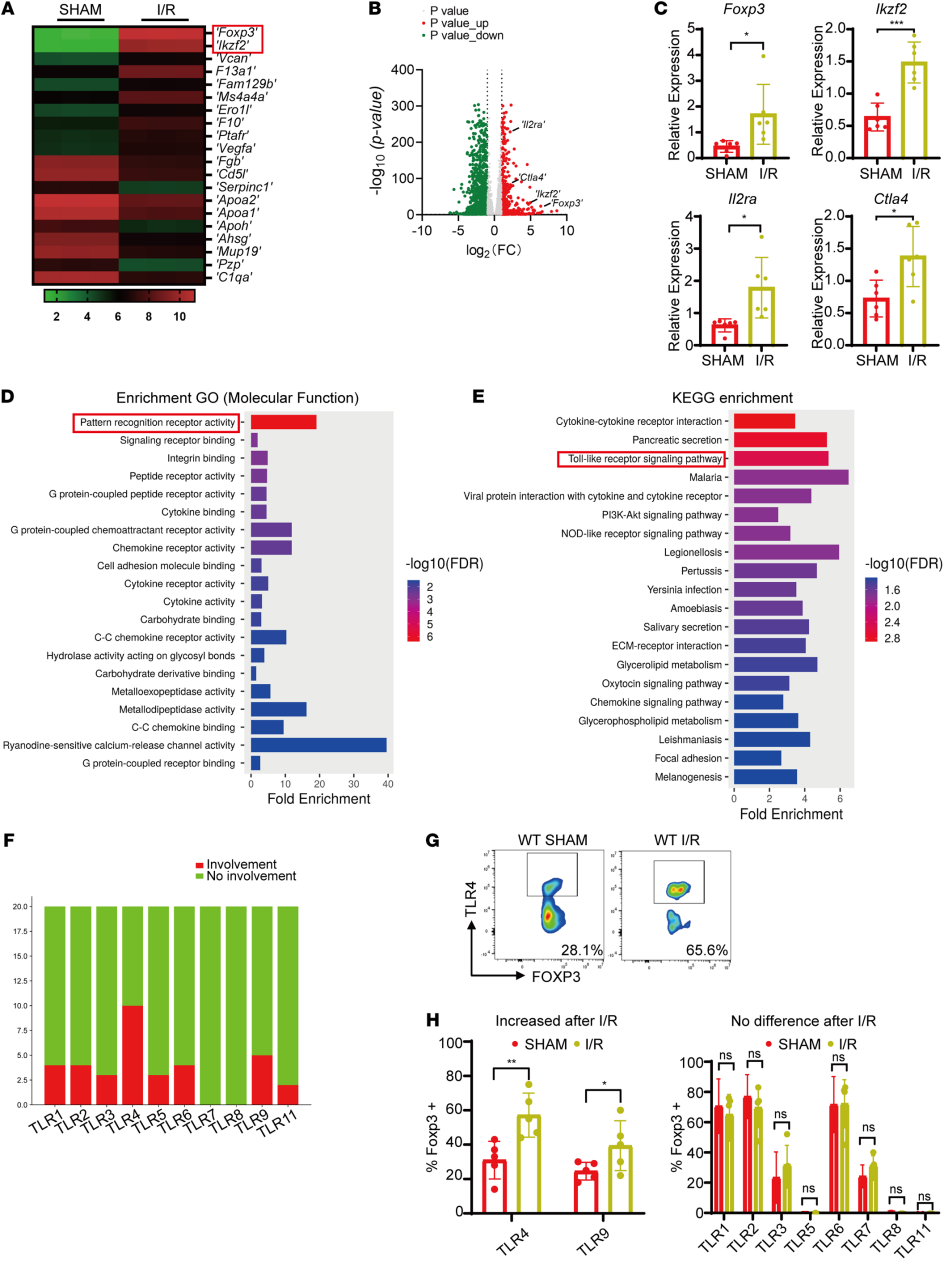
*Tlr4* is crucial for hepatic Tregs function following liver I/R. (**A**) Heatmap of DEGs (*P*_adj_ < 0.05) in hepatic Tregs from WT mice before and after liver I/R (*n* = 3 per group). (**B**) Volcano plot of DEGs in hepatic Tregs from WT mice before and after liver I/R. (**C**) Quantitative PCR analysis of *Foxp3, Ikzf2, Il2ra,* and *Ctla4* mRNA expression in Tregs from WT mice before and after liver I/R (*n* = 6 per group). (**D**) GO molecular function analysis of DEGs in hepatic Tregs. (**E**) KEGG pathway enrichment analysis of DEGs in hepatic Tregs. (**F**) IPA of DEGs in hepatic Tregs. (**G**) Representative FACS plots of TLR4 expression in hepatic Tregs before and after liver I/R. (**H**) Flow cytometric quantification of TLRs expression in hepatic Tregs from WT mice before and after liver I/R (*n* = 5 per group). Statistical analyses were performed using unpaired, 2-tailed *t* tests (**C** and **H**). *P < 0.05, **P < 0.01, ***P < 0.001. DEGs, differentially expressed genes; GO, Gene Ontology; KEGG, Kyoto Encyclopedia of Genes and Genomes; IPA, Ingenuity Pathways Analysis; TLR, Toll-like receptor.

To investigate the mechanisms by which hepatic Tregs protect against liver I/R injury, we performed bulk RNA-seq on hepatic Tregs isolated from WT mice before and after I/R. DEG analysis revealed 141 upregulated and 628 downregulated genes following I/R. Notably, *Foxp3* (28) and *Ikzf2* (29), both critical regulators of Treg immunosuppressive function, were among the genes with the most marked changes (Figure 2A). Additionally, key genes involved in Treg differentiation and function, including *Ctla4* (30) and *Il2ra* (31), were markedly upregulated in response liver I/R (Figure 2B). These transcriptional changes were validated by qPCR, which confirmed increased expression of these genes in hepatic Tregs from I/R mice compared with sham controls (Figure 2C). To further understand the regulatory pathways activated in hepatic Tregs after I/R, we conducted functional enrichment analyses. GO molecular function analysis showed marked enrichment in pattern recognition receptor (PRR) activity (Figure 2D), suggesting that liver I/R activated PRR-related signaling pathways in Tregs. KEGG pathway analysis further indicated robust enrichment of Toll-like receptor signaling pathways (Figure 2E), implicating a potential role for TLRs in modulating Treg function during I/R injury.

To identify specific TLRs involved, we performed the Ingenuity Pathways Analysis (IPA) of DEGs. Among the TLR family members, TLR4 emerged as the receptor most frequently associated with the top 20 signaling pathways (Figure 2F). Flow cytometry confirmed TLR4 as the most significantly upregulated TLR on Tregs following liver I/R (Figure 2, G and H). Together, these findings suggest that TLR4 plays a critical role in mediating Treg immunoregulatory function in the setting of liver I/R injury.

### Specific deletion of TLR4 in Tregs exacerbates liver I/R injury.

To investigate the role of TLR4 signaling specifically in Tregs during liver I/R injury, we generated Treg-specific TLR4 knock out (Treg-*Tlr4*^–/–^) mice by crossing *Tlr4*^fl/fl^ mice with *Foxp3-*Cre mice (Supplemental Figure 2A). Genotyping by PCR confirmed successful recombination, as indicated by the presence of loxp-flanked *TLR4* alleles and *Cre* recombinase expression (Supplemental Figure 2B). To validate TLR4 deletion in Tregs, CD4^+^FOXP3^–^ Tregs and CD4^+^FOXP3^–^ effector T cells (Teffs) were isolated via magnetic bead sorting from *Tlr4*^fl/fl^, *Tlr4*^–/–^, and Treg-*Tlr4*^–/–^ mice. PCR analysis showed the absence of *Tlr4* DNA in Tregs but not in Teffs from Treg-*Tlr4*^–/–^ mice (Supplemental Figure 2C). Immunofluorescence staining further confirmed TLR4 expression in both Teffs and Tregs in *Tlr4*^fl/fl^ mice, whereas TLR4 colocalized only with Teffs but not Tregs in Treg-*Tlr4*^–/–^ mice (Supplemental Figure 2D). These results validated the successful generation of the Treg-specific *Tlr4*-deficient mouse.

**Figure 3:**
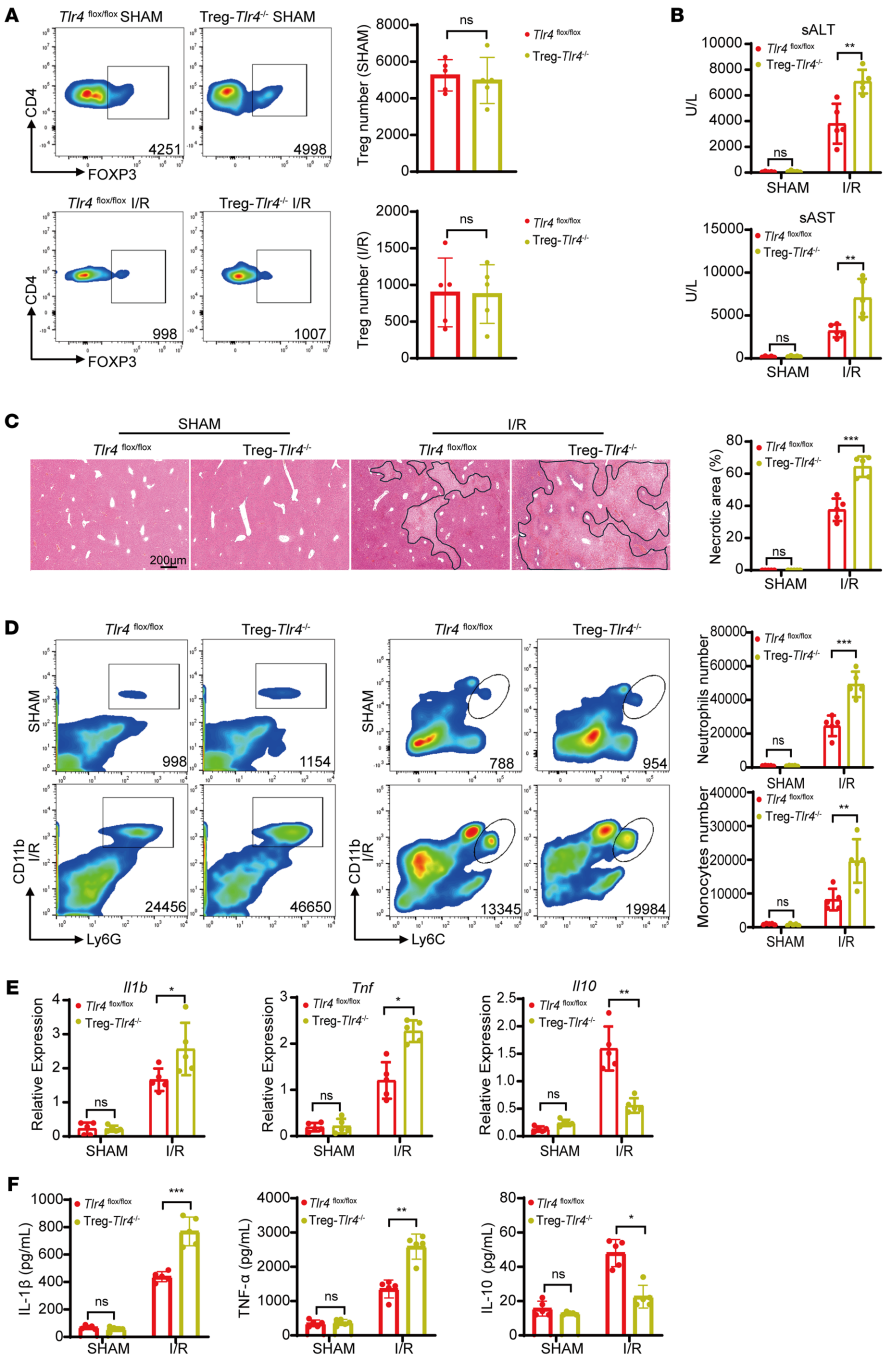
Specific deletion of *Tlr4* in Tregs exacerbates liver I/R injury. (**A**) Flow cytometry quantification of hepatic CD4^+^FOXP3^+^ Tregs in *Tlr4*^fl/fl^ and Treg-*Tlr4*^–/–^ mice before and after liver I/R (*n* = 5 per group). (**B**) Serum ALT and AST levels in *Tlr4*^fl/fl^ and Treg-*Tlr4*^–/–^ mice before and after liver I/R (*n* = 5 per group). (**C**) Representative H&E staining images of ischemia liver lobe from *Tlr4*^fl/fl^ and Treg-*Tlr4*^–/–^ mice before and after liver I/R (*n* = 5 per group). Scale bars: 200 μm. (**D**) Quantification of hepatic infiltrating CD11b^+^Ly6G^+^ neutrophils and CD11b^+^ Ly6C^+^ monocytes by flow cytometry in *Tlr4*^fl/fl^ and Treg-*Tlr4*^–/–^ mice before and after liver I/R (*n* = 5 per group). (**E**) Quantitative PCR analysis of *Il1b*, *Tnf*, and *Il10* mRNA expression in liver from sham and I/R *Tlr4*^fl/fl^ and Treg-*Tlr4*^–/–^ mice (*n* = 5 per group). (**F**) Serum IL-1β, TNF-α, and IL-10 levels from *Tlr4*^fl/fl^ and Treg-*Tlr4*^–/–^ mice before and after liver I/R (*n* = 5 per group). Statistical analyses were performed using unpaired, 2-tailed *t* tests (**A**) and 2-way ANOVA with Šidák’s post test (**B**–**F**). *P < 0.05, **P < 0.01, ***P < 0.001. ALT, Alanine aminotransferase; AST, Aspartate aminotransferase; I/R, ischemia/reperfusion.

To determine the functional consequence of TLR4 deletion in Tregs, Treg- *Tlr4*^/–^ mice and *Tlr4*^fl/fl^ control mice were subjected to liver I/R model. The frequency of CD4^+^FOXP3^+^ Tregs was comparable between *Tlr4*^fl/fl^ and Treg-*Tlr4*^–/–^ mice in both sham and I/R conditions (Figure 3A). However, following I/R, Treg-*Tlr4*^–/–^ mice exhibited significantly elevated serum levels of ALT and AST levels and greater hepatic necrosis compared with *Tlr4*^fl/fl^ controls (Figure 3, B and C). These mice also displayed increased infiltration of hepatic neutrophils and monocytes (Figure 3D). TNF-α, IL-1β, and IL-10 levels were minimal in both genotypes under sham conditions. In contrast, after I/R, Treg-*Tlr4^–/–^* mice exhibited significantly higher levels of TNF-α and IL-1β and lower levels of IL-10 compared with *Tlr4*^fl/fl^ mice in both the ischemic liver and serum (Figure 3, E and F). Collectively, these results identified that TLR4 expression in Tregs is essential for limiting liver inflammation and injury during I/R, supporting a key role for TLR4 in mediating Treg-dependent immunoprotection.

### TLR4 expression in Tregs alleviates liver I/R injury in Treg-depleted mice.

**Figure 4:**
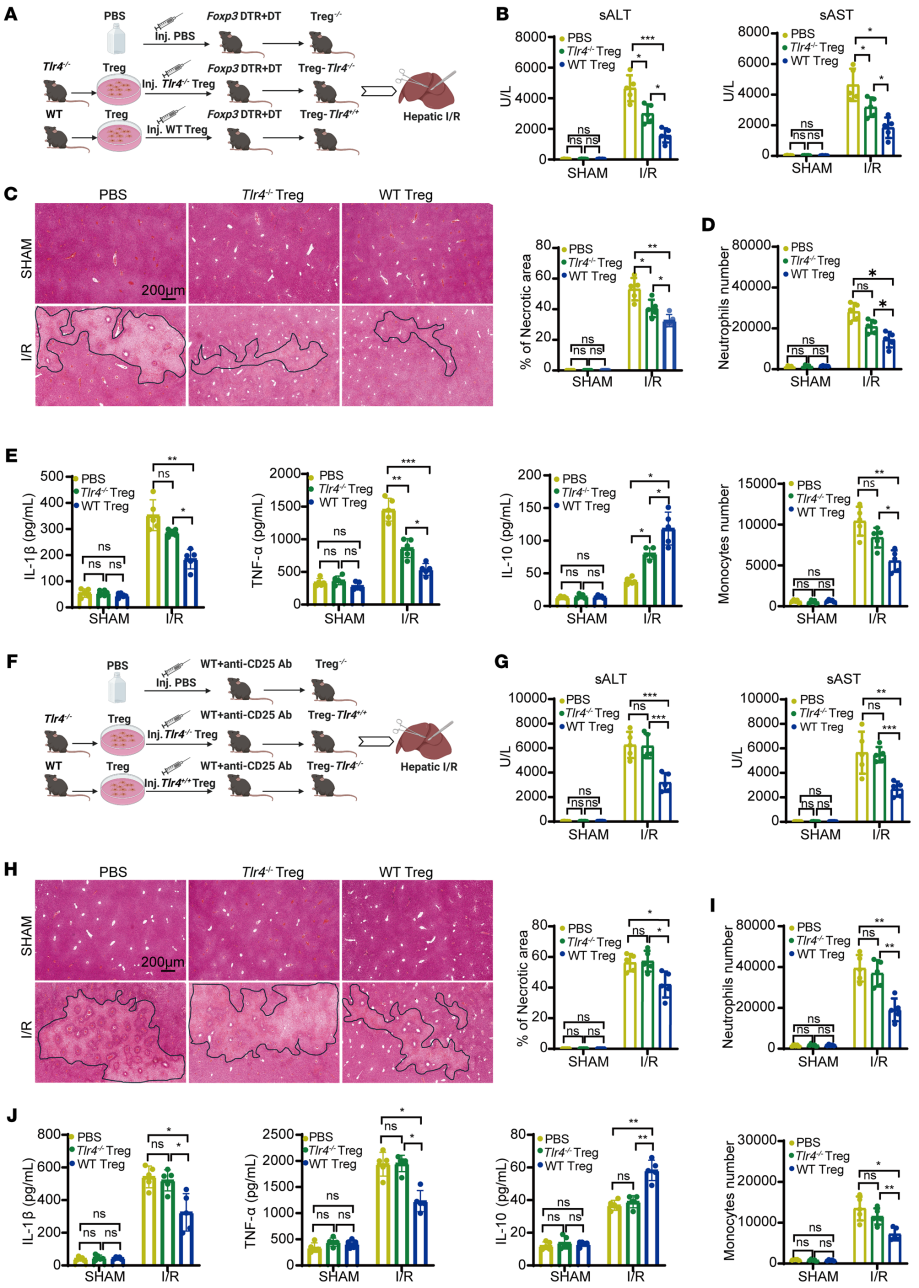
*Tlr4* expression in Tregs alleviates liver I/R injury in Treg-depleted mice. (**A**) Schematic diagram of the adoptive transfer experiment. DT-induced Treg depletion *Foxp3* DTR mice were administered PBS, *Tlr4*^–/–^ Tregs, or WT Tregs via tail vein injection 12 hours before liver I/R. (**B**) Serum ALT and AST levels in Treg-depleted *Foxp3* DTR mice administered PBS, *Tlr4*^–/–^ Tregs, or WT Tregs before and after liver I/R (*n* = 5 per group). (**C**) Representative H&E staining images of the ischemic liver in Treg-depleted *Foxp3* DTR mice administered PBS, *Tlr4*^–/–^ Tregs, or WT Tregs before and after liver I/R (*n* = 5 per group). Scale bars: 200 μm. (**D**) Quantification of hepatic infiltrating CD11b^+^Ly6G^+^ neutrophils and CD11b^+^Ly6C^+^ monocytes in Treg-depleted *Foxp3* DTR mice administered PBS, *Tlr4*^–/–^ Tregs, or WT Tregs before and after liver I/R (*n* = 5 per group). (**E**) Serum IL-1β, TNF-α, and IL-10 levels from Treg-depleted *Foxp3* DTR mice administered PBS, *Tlr4*^–/–^ Tregs, or WT Tregs before and after liver I/R (*n* = 5 per group). (**F**) Experimental design of adoptive transfer in anti-CD25 Ab-induced Treg-depleted WT mice administered PBS, *Tlr4*^–/–^ Tregs, or WT Tregs before and after liver I/R. (**G**) Serum ALT and AST levels in Treg-depleted WT mice were adoptively transferred with PBS, *Tlr4*^–/–^ Tregs, or WT Tregs before and after liver I/R (*n* = 5 per group). (**H**) Representative H&E staining images of the ischemic liver in Treg-depleted WT mice administered PBS, *Tlr4*^–/–^ Tregs, or WT Tregs before and after liver I/R (*n* = 5 per group). Scale bars: 200 μm. (**I**) Quantification of hepatic infiltrating CD11b^+^Ly6G^+^ neutrophils and CD11b^+^Ly6C^+^ monocytes in Treg-depleted WT mice administered PBS, *Tlr4*^–/–^ Tregs, or WT Tregs before and after liver I/R (*n* = 5 per group). (**J**) Serum IL-1β, TNF-α, and IL-10 levels from Treg-depleted WT mice adoptively transferred with PBS, *Tlr4*^–/–^ Tregs, or WT Tregs after I/R (*n* = 5 per group). Statistical analyses were performed using 1-way ANOVA with Tukey’s post test (**B**–**E** and **G**–**J**). *P < 0.05, **P < 0.01, ***P < 0.001. ALT, Alanine aminotransferase; AST, Aspartate aminotransferase; I/R, ischemia/reperfusion.

To further investigate the protective role of TLR4 in mediating anti-inflammatory effects of Treg, we performed adoptive transfer experiments using Treg-depleted *Foxp3* DTR mice. Tregs were isolated from donor mice using magnetic beads-based sorting (Supplemental Figure 3A). Recipient mice then received an intravenous injection of PBS, *Tlr4*^–/–^ Tregs, or WT Tregs 12 hours prior to liver I/R (Figure 4A). Adoptive transfer of WT Tregs significantly attenuated liver I/R injury, as indicated by lower serum ALT and AST levels compared with PBS- and *Tlr4*^–/–^ Treg-treated mice (Figure 4B). Histological analysis revealed smaller necrotic areas in livers of mice that received WT Tregs (Figure 4C). Furthermore, these mice exhibited diminished infiltration of neutrophils and monocytes, lower levels of pro-inflammatory mediators (TNF-α and IL-1β), elevated IL-10 expression in liver tissues and serum (Figure 4, D and E, and Supplemental Figure 3, B and C). We observed similar outcome in Treg-depleted WT mice treated with anti-CD25 antibody. Adoptive transfer of WT Tregs markedly mitigated liver I/R compared to adoptive transfer of *Tlr4*^–/–^ Tregs or the PBS control group, as evidenced by lower liver enzyme levels, reduced hepatic necrosis, and dampened inflammatory responses (Figure 4, F–J, and Supplemental Figure 3, D and E). These results reinforce the critical role of TLR4 in Treg-mediated protection against liver I/R injury and suggest that TLR4 signaling in Tregs is required for their anti-inflammatory function in vivo.

### TLR4 expression in Tregs mitigates liver I/R injury in Treg-Tlr4^–/–^ mice.

**Figure 5:**
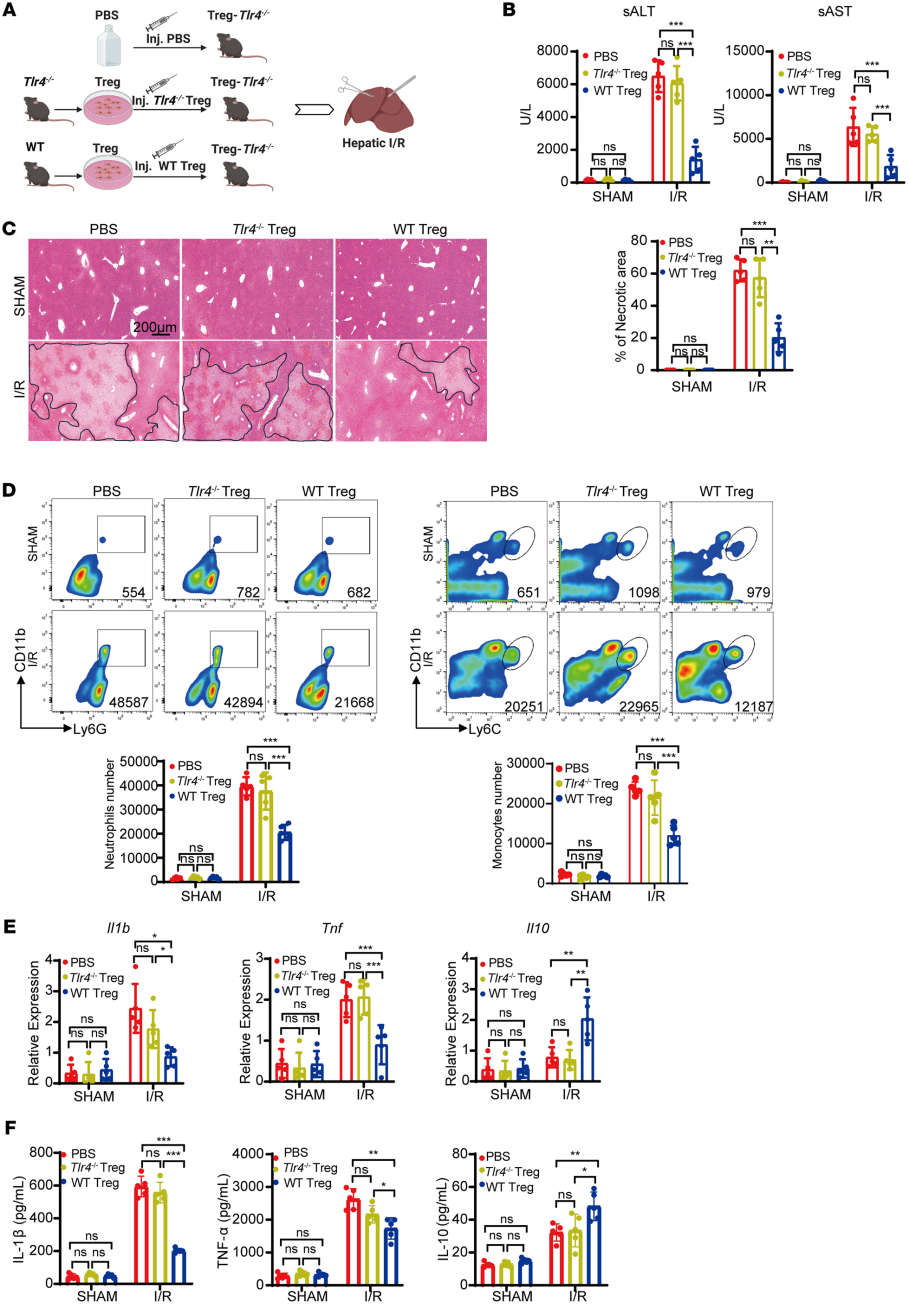
*Tlr4* expression in Tregs mitigates liver I/R injury in Treg-*Tlr4*^–/–^ mice. (**A**) Schematic diagram of the adoptive transfer experiment. Treg-*Tlr4*^–/–^ mice were administrated with PBS, *Tlr4*^–/–^ Tregs, or WT Tregs via tail vein injection 12 hours before I/R. (**B**) Serum ALT and AST levels in the 3 groups of mice before and after I/R (*n* = 5 per group). (**C**) Representative H&E staining images of ischemia liver lobes in the 3 groups of mice before and after I/R (*n* = 5 per group). Scale bars: 200 μm. (**D**) Quantification of hepatic infiltrating CD11b^+^Ly6G^+^ neutrophils and CD11b^+^Ly6C^+^ monocytes by flow cytometry in the 3 groups before and after I/R (*n* = 5 per group). (**E**) Quantitative PCR analysis of *Il1b*, *Tnf*, and *Il10* mRNA expression in ischemia liver lobes in the 3 groups of mice before and after I/R (*n* = 5 per group). (**F**) Serum IL-1β, TNF-α, and IL-10 levels in the 3 groups of mice before and after I/R (*n* = 5 per group). Statistical analyses were performed using 1-way ANOVA with Tukey’s post test (**B**–**F**). *P < 0.05, **P < 0.01, ***P < 0.001. ALT, Alanine aminotransferase; AST, Aspartate aminotransferase; I/R, ischemia/reperfusion; Tregs, regulatory T cells.

To determine whether adoptive transfer of exogenous Tregs could rescue the exacerbated I/R injury resulting from TLR4 deficiency in Tregs, Treg-*Tlr4*^–/–^ mice were administered PBS, *Tlr4*^–/–^ Tregs or WT Tregs 12 hours prior to liver I/R (Figure 5A). Mice receiving WT Tregs exhibited significantly lower serum ALT and AST levels compared to those treated with *Tlr4*^–/–^ Tregs or PBS following I/R (Figure 5B). Histological and flow cytometry analysis revealed reduced hepatic necrosis and decreased infiltration of neutrophils and monocytes in WT Treg-treated mice (Figure 5, C and D). Moreover, the hepatic proinflammatory mediators TNF-α and IL-1β were markedly decreased, while anti-inflammatory IL-10 levels were elevated in the WT Treg-treated group compared with other treatment groups in liver and serum (Figure 5, E and F). These results indicate that WT Tregs can compensate for the loss of TLR4 signaling in Tregs, restoring their antiinflammatory function and attenuating liver I/R injury. This finding supports the therapeutic potential of adoptive WT Treg transfer as a strategy to mitigate liver I/R injury, particularly in settings of impaired Treg function.

### Tlr4 deficiency in Tregs impairs Il10 production by disrupting the Myd88/cAMP signaling pathway following liver I/R.

**Figure 6:**
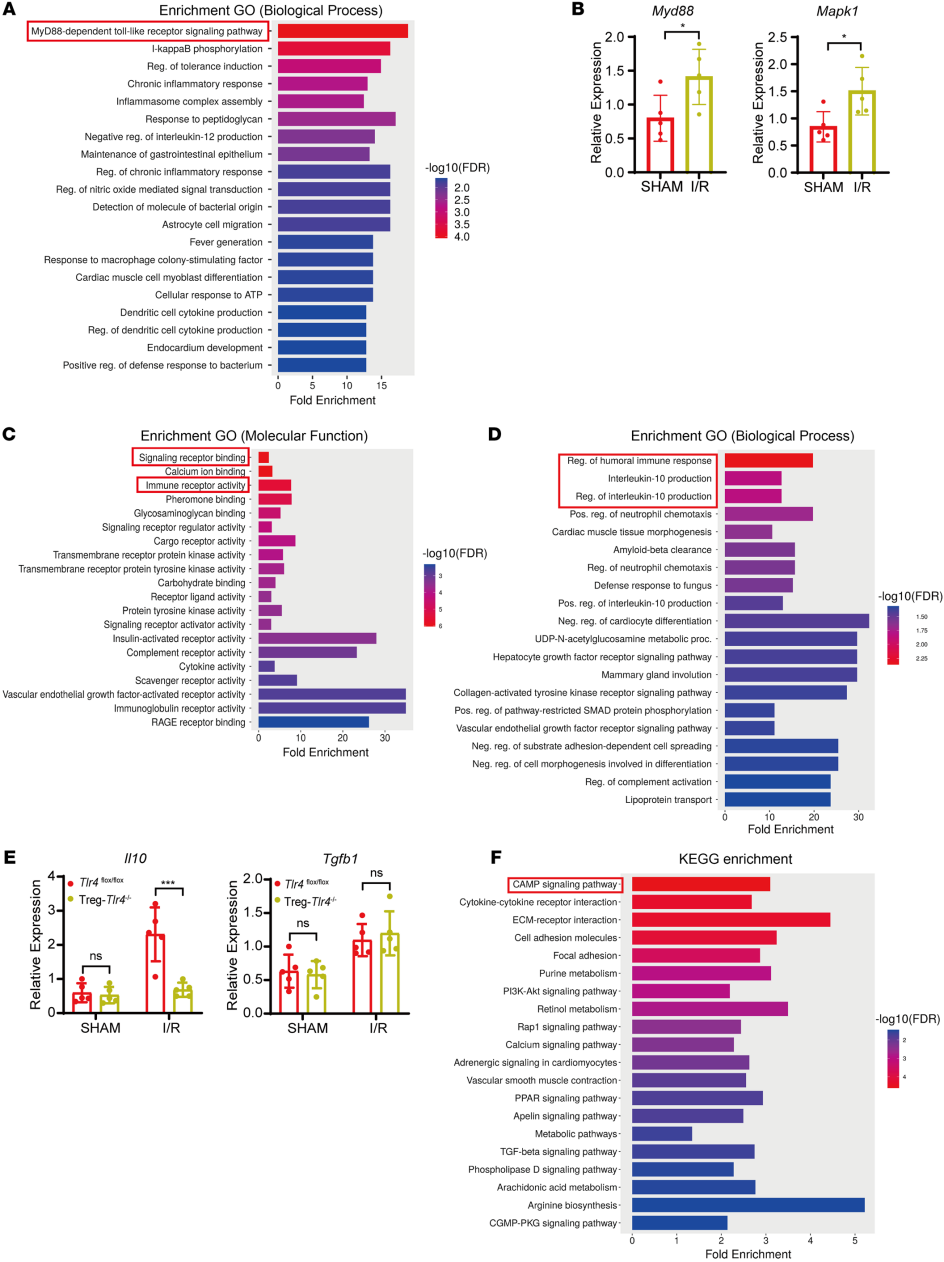
*Tlr4* deficiency in Tregs impairs *Il10* production by disrupting the *Myd88/cAMP* signaling pathway following liver I/R. (**A**) GO analysis of biological processes in WT Tregs before and after liver I/R. (**B**) Quantitative PCR analysis of *Myd88* and *Mapk1* mRNA expression in Tregs from sham and I/R WT mice (*n* = 5 per group). (**C**) GO analysis of molecular functions of DEGs in hepatic Tregs isolated from *Tlr4*^fl/fl^ mice and Treg-*Tlr4*^–/–^ mice following liver I/R. (**D**) GO analysis of biological processes of DEGs in hepatic Tregs isolated from *Tlr4*^fl/fl^ mice and Treg-*Tlr4*^–/–^ mice following liver I/R. (**E**) Quantitative PCR analysis of *Il10* and *Tgfb1* mRNA expression in Tregs from *Tlr4*^fl/fl^ mice and Treg-*Tlr4*^–/–^ mice (*n* = 5 per group). (**F**) KEGG pathway enrichment analysis of DEGs in hepatic Tregs isolated from *Tlr4*^fl/fl^ mice and Treg-*Tlr4*^–/–^ mice following liver I/R. Statistical analyses were performed using unpaired, 2-tailed *t* tests (**B**) and 2-way ANOVA with Šidák’s post test (**E**). *P < 0.05, ***P < 0.001. DEGs, differentially expressed genes; GO, Gene Ontology; KEGG, Kyoto Encyclopedia of Genes and Genomes.

To investigate the mechanisms by which TLR4 regulates the immunosuppressive function of Tregs, we performed GO analysis of biological processes in WT Tregs before and after liver I/R. Among the top enriched GO terms was the MyD88-dependent Toll-like receptor signaling pathway (Figure 6A), suggesting a role for MyD88 signaling in Treg-mediated protection. This was further supported by qPCR, which showed increased expression of genes associated with this pathway, including *Myd88* and *Mapk1* in WT Tregs following I/R (Figure 6B).

We next conducted bulk RNA-seq on hepatic Tregs isolated from *Tlr4*^fl/fl^ and Treg-*Tlr4*^–/–^ mice following I/R. GO molecular function analysis revealed an enrichment in signaling receptor binding and immune receptor activity, pointing to altered responsiveness of Tregs to immune signals due to *Tlr4* deficiency (Figure 6C). Additionally, GO analysis of biological process analysis identified marked enrichment for terms related to IL-10 production (Figure 6D), suggesting that *Tlr4* deficiency may impair Treg function via downregulation of IL-10. Given Tregs’ role in secreting IL-10 and TGF-β to suppress inflammation, we quantified their expression in hepatic Tregs from *Tlr4*^fl/fl^ and Treg-*Tlr4*^–/–^ mice. Compared with *Tlr4*^fl/fl^ controls, Treg-*Tlr4*^–/–^ mice exhibited significantly lower *Il10* mRNA levels following I/R, whereas *Tgfb1* expression remained unchanged (Figure 6E). KEGG pathway analysis further revealed substantial enrichment in cyclic AMP (cAMP) signaling pathway, which is known to promote antiinflammatory responses (32) and enhance MyD88-dependent IL-10 production (33) (Figure 6F). Together, these findings suggested that *Tlr4* deficiency compromises the immunosuppressive capacity of Tregs during liver I/R injury by impairing *Myd88* and *cAMP* signaling pathways, leading to reduced *Il10* production.

### TLR4/MyD88/ERK/CREB-mediated IL-10 production in Tregs attenuates liver I/R injury.

A previous study has shown that cAMP regulates IL-10 production in normal human T lymphocytes and macrophages (34). The cAMP-response element-binding protein (CREB), a key transcription factor downstream of cAMP signaling, can bind to the *Il10* promoter and enhance IL-10 production in macrophages (35). Moreover, TLR4 activation can induce ERK and p38 MAPK phosphorylation, which, in turn, promotes CREB activation (36). Based on these findings, we hypothesized that TLR4 signaling in Tregs might trigger these MAPK pathways (ERK or p38), affecting CREB phosphorylation and enhancing IL-10 production.

**Figure 7:**
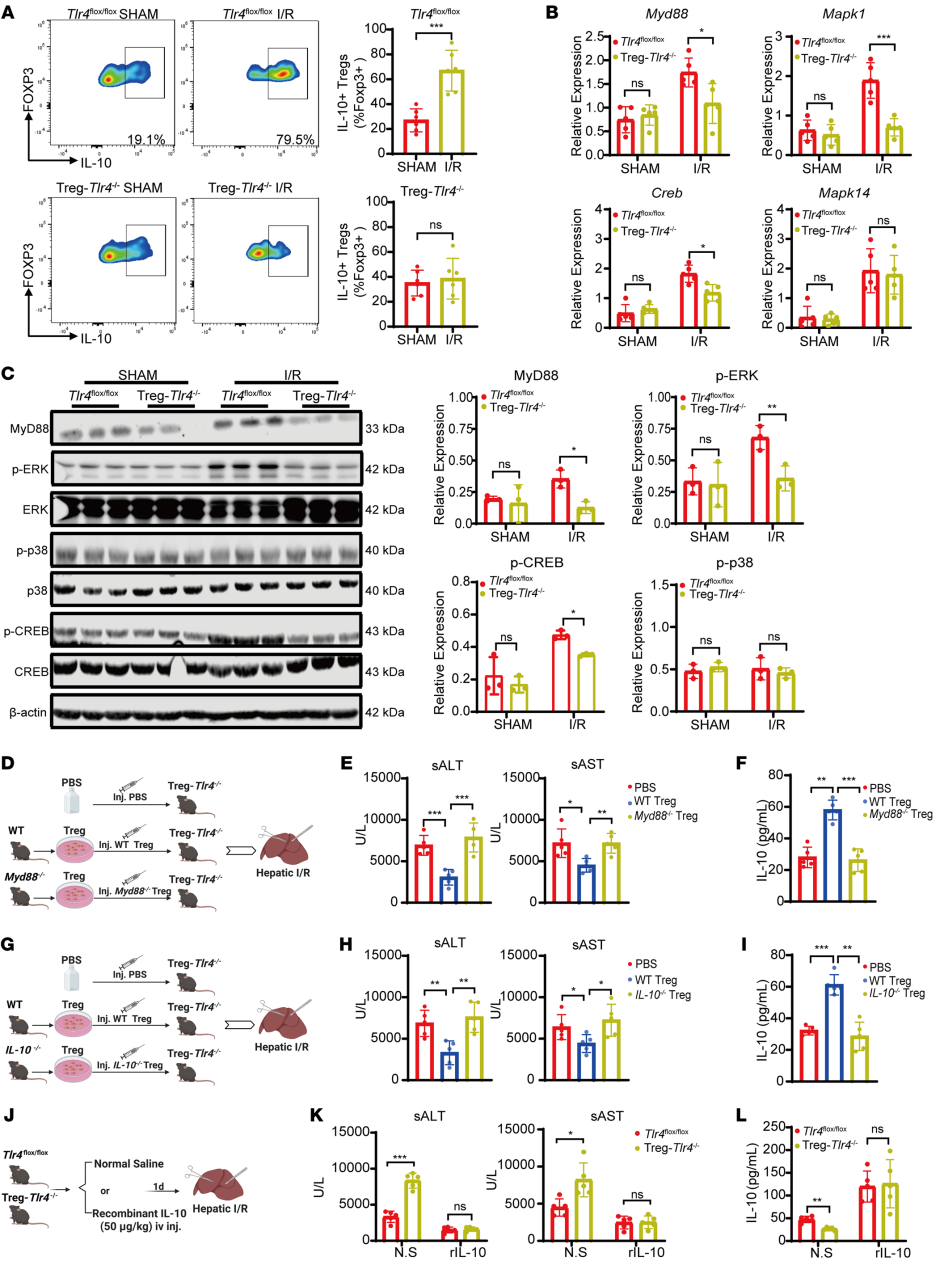
TLR4/MyD88/ERK/CREB-mediated IL-10 production in Tregs alleviates liver I/R injury. (**A**) Quantification of IL-10 expression in Tregs by flow cytometry from *Tlr4*^fl/fl^ mice and Treg-*Tlr4*^–/–^ mice before and after I/R (*n* = 6 per group). (**B**) Quantitative PCR analysis of *Myd88, Mapk1, Mapk14*, and *Creb* mRNA expression in Tregs from *Tlr4*^fl/fl^ mice and Treg-*Tlr4*^–/–^ mice before and after I/R (*n* = 5 per group). (**C**) Western blot analysis of MyD88, p-ERK, p-p38, and p-CREB protein expression levels in Tregs from *Tlr4*^fl/fl^ mice and Treg-*Tlr4*^–/–^ mice before and after I/R (*n* = 3 per group). (**D**) Experimental design for adoptive transfer of PBS, WT Tregs, or *Myd88*^–/–^ Tregs into Treg-*Tlr4*^–/–^ mice via tail vein injection 12 hours before I/R. (**E**) Serum ALT and AST levels in Treg-*Tlr4*^–/–^ mice receiving PBS, WT Tregs, or *Myd88*^–/–^ Tregs after liver I/R (*n* = 5 per group). (**F**) Serum IL-10 levels from Treg-*Tlr4*^–/–^ mice adoptively transferred with PBS, *Myd88*^–/–^ Tregs or WT Tregs after liver I/R (*n* = 5 per group). (**G**) Experimental design for adoptive transfer of PBS, WT Tregs, or *IL-10*^–/–^ Tregs into Treg-*Tlr4*^–/–^ mice via tail vein injection 12 hours before I/R. (**H**) Serum ALT and AST levels in Treg-*Tlr4*^–/–^ mice receiving PBS, WT Tregs, or *IL-10*^–/–^ Tregs after liver I/R (*n* = 5 per group). (**I**) Serum IL-10 levels from Treg-*Tlr4*^–/–^ mice adoptively transferred with PBS, *IL-10*^–/–^ Tregs or WT Tregs after liver I/R (*n* = 5 per group). (**J**) Experimental design for administration of recombinant IL-10 and its control into Treg-*Tlr4*^–/–^ mice. (**K**) Serum ALT and AST levels in *Tlr4*^fl/fl^ mice and Treg-*Tlr4*^–/–^ mice receiving recombinant IL-10 or its control after I/R (*n* = 5 per group). (**L**) Serum IL-10 levels from Treg-*Tlr4*^–/–^ mice receiving recombinant IL-10 or its control after I/R (*n* = 5 per group). Statistical analyses were performed using unpaired, 2-tailed *t* tests (**A**), 2-way ANOVA with Šidák’s post test (**B**, **C**, **K**, and **L**) and 1-way ANOVA with Tukey’s post test (**E**, **F**, and **H**–**I**). *P < 0.05, **P < 0.01, ***P < 0.001. ALT, Alanine aminotransferase; AST, Aspartate aminotransferase; I/R, ischemia/reperfusion; rIL-10, recombinant IL-10.

To test this, we measured IL-10 levels in hepatic Tregs from *Tlr4*^fl/fl^ and Treg-*Tlr4*^/–^ mice before and after liver I/R. In *Tlr4*^fl/fl^ mice, IL-10 expression was significantly increased in Tregs following liver I/R, whereas no such increase was observed in Treg-*Tlr4*^–/–^ mice (Figure 7A). Furthermore, MyD88, phosphorylated ERK (p-ERK), and phosphorylated CREB (p-CREB) mRNA and protein levels were markedly lower in Tregs from Treg-*Tlr4*^–/–^ mice compared with *Tlr4*^fl/fl^ controls after liver I/R. In contrast, phosphorylated p38 levels showed no significant differences between these mice (Figure 7, B and C). Adoptive transfer of WT Tregs, but not *Tlr4*^–/–^ Tregs, restored MyD88, p-ERK, and p-CREB protein expression in Treg-*Tlr4*^–/–^ mice after liver I/R (Supplemental Figure 4A).

To further confirm involvement of the MyD88/ERK/CREB signaling pathway in IL-10 production in hepatic Tregs and liver protection, we adoptively transferred *Myd88*^–/–^or *IL-10*^–/–^ Tregs into Treg-*Tlr4*^–/–^ mice (Figure 7, D and G). Neither *Myd88*^–/–^ nor *IL-10*^–/–^ Tregs transfer reduced ALT and AST levels, limited hepatic necrosis, increased IL-10 expression, and attenuated inflammatory in Treg-*Tlr4*^–/–^ mice (Figure 7, E, F, H, and I, and Supplemental Figure 4, B–E), indicating that both MyD88 and IL-10 are essential for Treg-mediated protection.

Finally, to determine whether exogenous IL-10 could compensate for Treg-derived IL-10 deficiency and Treg-mediated protection, recombinant IL-10 was administered via tail vein injection to *Tlr4*^fl/fl^ and Treg-*Tlr4*^–/–^ mice 24 hours prior to liver I/R (Figure 7J). Exogenous IL-10 significantly reduced ALT and AST levels, diminished hepatic necrosis, and attenuated inflammation in both *Tlr4*^fl/fl^ and Treg-*Tlr4*^–/–^ mice (Figure 7, K and L, and Supplemental Figure 4, F and G). To further assess whether the protective effect of exogenous IL-10 depends on the presence of Tregs, we administered recombinant IL-10 to both Treg-sufficient and Treg-depleted mice (using *Foxp3* DTR mice with or without DT pretreatment) subjected to hepatic I/R. Exogenous IL-10 partially ameliorated liver injury even in the absence of Tregs, though the degree of protection was less pronounced than that observed in Treg-sufficient mice receiving recombinant IL-10 treatment (Supplemental Figure 4, H and I). Together, these results demonstrate that TLR4 signaling in Tregs enhances IL-10 secretion through the MyD88/ERK/CREB signaling pathway, thereby attenuating liver I/R injury.

## Discussion

Liver I/R injury remains a significant clinical challenge in hepatic surgeries, often contributing postoperative complications and transplant failures (3, 37). In this study, we identified a protective role for TLR4 signaling in Tregs during liver I/R injury. Although hepatic Treg numbers decreased following I/R, the remaining Tregs exhibited enhanced immunosuppressive activity. Our data identify a Treg-intrinsic role for TLR4 in the early response to hepatic ischemia reperfusion and clarify how this differs from TLR4 signaling in hepatocytes and KCs. Whereas TLR4 engagement in parenchymal and resident myeloid cells drives cytokine release and tissue injury, TLR4 in Tregs is required to sustain their regulatory program in the acute phase. Treg-restricted deletion of TLR4 reduced ERK and CREB activation, lowered IL-10 production, and diminished hepatoprotection after adoptive transfer, while recombinant IL-10 restored protection. These findings indicate that TLR4 acts as a licensing signal in Tregs that preserves Foxp3-guided function and IL-10 output when Tregs are limiting in the postischemic liver, in contrast with the injury-promoting effects of TLR4 in hepatocytes and KCs. Together with reports that TLR4 engagement can alter Treg stability and the Th17 and Treg balance in other sterile injury contexts, our results support a cell type–specific model in which the net impact of TLR4 on liver injury reflects the sum of proinflammatory signaling in parenchymal and myeloid compartments and a protective, program-sustaining signal in Tregs. This distinction should inform therapeutic strategies that aim to blunt detrimental TLR4 activity without impairing Treg-dependent regulation.

Previous studies have suggested that modulating the Th17/Treg ratio may offer therapeutic benefit in liver I/R injury (38, 39). However, direct in vivo evidence of Tregs’ protective benefit in liver I/R injury has been limited. By combining patient samples and clinic-relevant murine models, we demonstrated that hepatic Treg numbers inversely correlate with liver injury severity and that Treg depletion exacerbated liver injury, providing robust support for the immunoprotective role of Tregs during liver I/R injury.

Although Tregs commonly increase over time in chronic inflammatory settings (40–42), acute ischemic injury produces abrupt hypoxia reoxygenation and oxidative stress that can transiently reduce local Tregs (43, 44), together with the rapid recruitment of neutrophils and inflammatory monocytes (45, 46). Our absolute-count analyses at 6 hours after reperfusion are consistent with this early phase, during which Tregs become rate limiting for IL-10–mediated protection via the TLR4/MyD88/ERK/CREB pathway. Transcriptomic analysis of hepatic Tregs revealed upregulation of genes associated with immunosuppressive function following I/R, and enrichment of TLR signaling pathways. Our RNA-seq data identified the involvement of multiple TLRs, both surface (*Tlr1*, *Tlr2*, *Tlr4*, *Tlr5*, and *Tlr6*), and intracellular (*Tlr3*, *Tlr7*, *Tlr8*, and *Tlr9*), in the transcriptional regulation of Tregs during liver I/R. These TLRs are known to recognize distinct pathogen- or damage-associated molecular patterns; for instance, TLR1/2/6 respond to bacterial lipopeptides, enabling the immune system to combat bacteria by detecting unique lipid structures on these pathogens (47, 48). TLR5 detects flagellin, initiating immune responses against bacterial invasion (49), and TLR3/7/8/9 sense viral or bacterial nucleic acid (50, 51). Among these, TLR4 showed the most pronounced upregulation in hepatic Tregs by flow cytometry, consistent with its established role as a central pattern recognition receptor in both innate and adaptive immunity (52). This led us to focus on dissecting the role of TLR4 in Tregs under the sterile inflammatory context of liver I/R injury.

At baseline, T cells, including Tregs, can express TLR4 at low levels, with expression and function becoming more evident under inflammatory cues, this has been shown in primary CD4^+^ T cells and in Tregs in vivo (53). During hepatic I/R injury, the local milieu shifts within hours to hypoxia-reoxygenation and DAMP release. Both hypoxia signaling and DAMP exposure are known to upregulate TLR4 transcription or surface availability in inflamed tissues: HIF-1α can modulate TLR4 pathways under hypoxic stress, and disulfide-HMGB1, a prototypic reperfusion-phase DAMP, signals through TLR4 in myeloid cells and promotes inflammatory reprogramming. These mechanisms provide a biologically plausible route for rapid TLR4 upregulation on Tregs during the short I/R window (54, 55). Interestingly, our findings challenge the conventional view of TLR4 as solely a proinflammatory receptor (56, 57). In the context of Tregs, TLR4 activation initiates a cascade of intracellular signaling events, culminating in increased production of IL-10. Previous studies have shown that TLR4-MyD88 signaling induces IL-10 through the MEK/ERK/RSK and p38/CREB axes in macrophages (35), and that the cAMP pathway can further amplify MyD88-driven IL-10 expression in response to LPS (33). In line with these reports, we demonstrated that TLR4-mediated activation of the MyD88-dependent signaling pathway regulates the cAMP signaling pathway, thereby enhancing and sustaining IL-10 production.

While IL-10 was selectively reduced, TGF-β remained unchanged in our TLR4-deficient settings, this selective cytokine profile aligns with distinct molecular pathways governing IL-10 transcription and TGF-β activation. In T cells, including Tregs, IL-10 is driven by STAT3-centered programs and the Blimp-1/c-Maf axis, which codominate *Il10* transcription across CD4^+^ subsets (58–61). By contrast, TGF-β is frequently regulated posttranscriptionally and cell extrinsically; latent TGF-β requires activation via αvβ6 or αvβ8 integrins expressed on epithelial cells, dendritic cells, and effector Tregs, so steady-state transcript or total protein levels may remain stable even when local activation dynamics change (62–66). Moreover, TLR4 signaling can reshape immune programs without uniformly altering T cell–derived TGF-β, reported TLR4/TGF-β crosstalk is most extensively characterized in innate compartments, such as dendritic cells and macrophages, rather than through direct modulation of Treg-intrinsic TGF-β output (67). Consistent with these mechanisms, our in vivo data show that IL-10 decreases with Treg depletion and is restored by adoptive transfer of WT Tregs. Notably, at 6 hours after IR, the serum IL-10 level in Treg-*Tlr4*^–/–^ mice was significantly lower than that in *Tlr4*^fl/fl^ mice, supporting a Treg-intrinsic, TLR4-linked IL-10 program rather than a generalized suppression of immunoregulatory mediators.

Functionally, adoptive transfer of WT Tregs, but not *Tlr4*^–/–^ Tregs, into Treg-depleted mice significantly mitigated liver I/R injury. However, *Tlr4*^–/–^ Tregs still retained partial protective capacity, suggesting that other Treg-associated molecules contribute to their immunoregulatory effects. Our data indicate that, in addition to TLR4, TLR9 expression increases in hepatic Tregs at 6 hours after I/R. Moreover, activation of TLR9 suppresses Th17 cells and induces antiinflammatory IL10^+^ Tregs (68), raising the possibility that TLR9-dependent signaling and potentially other pattern-recognition pathways provide TLR4-independent compensation in this acute milieu. Moreover, several core Treg suppressive programs do not strictly require TLR4, including CTLA-4–mediated modulation of CD80/CD86 (69, 70) and high-affinity IL-2 consumption via CD25 (71). These mechanisms could remain at least partly intact in *Tlr4*^–/–^ Tregs and, thus, contribute to measurable benefit upon adoptive transfer. We also note that IL-10 production is reduced but not abolished in *Tlr4*^–/–^ Tregs in our model, so residual IL-10 may still aid hepatoprotection. Finally, trafficking/survival cues (e.g., chemokine receptors and tissue retention signals) are not expected to be uniformly impaired by TLR4 deletion and may allow *Tlr4*^–/–^ Tregs to reconstitute the regulatory niche after depletion. Together, these factors provide a biologically plausible basis for the partial, but clearly inferior, protection observed with *Tlr4*^–/–^ Tregs relative to WT Tregs.

Adoptive Treg transfer has shown promise in early phase clinical trials. A phase I clinical trial in liver transplantation demonstrated that autologous Treg transfer is safe, expands circulating Tregs, and reduces antidonor T cell responses (72). Previous research has identified that TLR4 can enhance Treg proliferation in vitro (73). Our findings provide additional mechanistic rationale for Treg-based therapy and suggest that engineering Tregs to enhance TLR4 expression, such as via chimeric antigen receptor (CAR) technology, may further improve their immunosuppressive capacity and persistence in vivo, thereby offering therapeutic benefit in liver surgery and transplantation.

Despite these insights, our study has some limitations. The isolation of Tregs using beads offers several advantages, including convenience, rapid processing, and the ability to yield sufficient quantities of viable cells. This method is particularly useful in applications such as adoptive transfer experiments, where maintaining high cell viability is crucial. However, it is important to note that beads-based Treg cell isolation may result in slightly lower purity compared to fluorescence-activated cell sorting. To mitigate this limitation and enhance the reliability and validity of experimental outcomes, in future experimental endeavors, meticulous attention should be devoted to minimize contamination from other cell types and improve the purity of isolated Treg populations. Furthermore, Tregs regulate immunity not only through cytokine secretion but also via direct cell-cell contact. The impact of TLR4 activation on Treg interactions with other immune cells or hepatocytes remains to be determined. Additionally, we recognize that the size of our human cohort (*n* = 16) and several mouse experiments (*n* = 5–6 per group) is limited. To address this limitation, key experiments were independently replicated at least 3 times with concordant results, and group sizes were prospectively defined based on established protocols in hepatic I/R models and were adequately powered for our primary endpoints (biochemical injury, histological changes, and flow-cytometric readouts). Nonetheless, larger cohorts will be necessary to refine effect size estimates, improve confidence interval precision, and enhance generalizability, and we plan to validate these findings in expanded patient cohorts and through additional preclinical repeats in future work.

In summary, our data demonstrate that the TLR4/MyD88/ERK/CREB axis in Tregs promotes IL-10 expression, thereby limiting liver I/R injury. These findings not only enhance our understanding of Treg biology in hepatic inflammation but also suggest that targeting TLR4 signaling in Tregs may offer a therapeutic strategy for managing surgical liver injury.

## Methods

### Sex as a biological variable.

Our study exclusively examined male mice, since estrogen influences immune response to liver I/R process (74). Liver samples from both men and women were included in this study. There was no bias in the grouping, and there were consistent results for both men and women.

### Human samples.

The study enrolled 16 patients diagnosed with hepatic hemangioma who underwent surgery at Wuhan Union Hospital. Exclusion criteria for the study included a history of chronic viral hepatitis, pregnancy, end-stage liver disease, or other known liver conditions that could influence liver function.

### Animals.

*C57BL/6J* male mice were obtained from Vital River (Beijing, China) and The Jackson Laboratory (Stock #: 000664). *Tlr4*^fl/fl^ mice (Stock #: 024872); *Foxp3*-Cre mice (Stock #: 016961); *MyD88*^–/–^ mice (Stock #: 009088); *Tlr4*^–/–^ mice (Stock #: 029015) and *IL-10* ^–/–^ mice (Stock #: 002251) were purchased from The Jackson Laboratory. Treg-*Tlr4*^–/–^ mice were obtained by crossing *Tlr4*^fl/fl^ mice with *Foxp3*-Cre mice. The environment was controlled with a 12-hour light/dark cycle, 60% ± 10% humidity, and a temperature of 23°C ± 2°C, with food and water provided ad libitum. Male mice underwent a segmental (70%) hepatic I/R procedure, as previously described (75). Briefly, anesthesia was induced via intraperitoneal injection of sodium ketamine (100 mg/kg) and xylazine (10 mg/kg). A midline laparotomy was performed to fully expose the operative field, with the middle and right liver lobes carefully lifted. Clamping of the hepatic portal (hepatic artery, portal vein, bile ducts) using a microvascular clamp leads to ischemia (Fine Science Tools, North Vancouver, BC, Canada) for 60 minutes. During the ischemic period, the mice were maintained at a consistent temperature of 32°C using a warming incubator chamber. Sham-operated controls underwent anesthesia, laparotomy, and portal triad exposure without the induction of ischemia. After a 6-hour reperfusion period, the mice were reanesthetized with inhaled isoflurane and euthanized via exsanguination. To administer recombinant IL-10, mice were injected with 50 μg/kg of recombinant IL-10 (76) or equivalent volume of saline 24 hours before surgery.

### Tregs in vivo elimination.

Diphtheria toxin (DT) or anti-mouse CD25 antibody were used for Treg depletion. *Foxp3* DTR mice were administered intraperitoneal injections of DT at a dose of 500 ng on 2 consecutive days before liver I/R (26). Anti-CD25 antibodies were administered intraperitoneally to WT mice at a dose of 300 μg per mouse for 2 consecutive days before liver I/R (27).

### Isolation and injection of Tregs.

The mouse spleen was harvested and dissociated to obtain a single-cell suspension. CD4^+^CD25^+^ Tregs were isolated using the CD4^+^CD25^+^ Regulatory T Cell Isolation Kit (MACS, 130091041) according to the manufacturer’s protocol. A total of 1 × 10^6^ Tregs were administered to mice via tail vein injection 12 hours before liver I/R (77).

### IHC.

Frozen human liver tissue sections were equilibrated to room temperature, hydrated in water for 3–5 minutes, and washed 3 times in PBS. Sections were blocked with 3% BSA or serum and incubated overnight at 4°C with anti-FOXP3 antibody (Supplemental Table 2) (40). After washing, sections were incubated with an HRP-labeled secondary antibody for 50 minutes, followed by DAB staining for color development, monitored microscopically. A minimum of 10 nonoverlapping, high-power fields (HPFs) per sample were randomly selected for quantification. FOXP3^+^ nuclei were quantified manually by our team members who were blinded to the experimental groups to ensure unbiased assessment.

### Isolation of hepatic nonparenchymal cells.

Hepatic nonparenchymal cells *(*NPCs) were isolated as previously described (78). Briefly, mouse livers were perfused via the portal vein with a 0.05% collagenase IV solution (1 mg/mL) at 37°C and a flow rate of 99 mL/h. Gentle massaging of the liver edges was performed during perfusion to ensure even distribution of the collagenase. The liver suspensions were centrifuged at 300*g* for 10 minutes at room temperature. The supernatant was collected and mixed with an OptiPrep separating solution (OptiPrep: PBS at a ratio 1:2). The mixture underwent density gradient centrifugation at 400*g* for 16 minutes at 22°C to isolate hepatic NPCs.

### Flow cytometry.

2 × 10^6^ NPCs were isolated from the liver as described previously. For the detection of intracellular IL-10 in Treg cells, single-cell suspensions were first stimulated with 2 μL Cell Activation Cocktail (Biolegend, Cat:423303) (including 40.5 μM phorbol-12-myristate 13-acetate, 669.3 μM ionomycin, and 2.5 mg/ml Brefeldin A) for 5 hours at 37°C under 5% CO_2_. Mouse Fc receptors were blocked by incubating cells with anti-Mouse CD16/CD32 in PBS for 30 minutes at 4°C. NPCs were incubated with fixable viability dye in PBS for 30 minutes at 4°C. Next, cells were surface stained, followed by intracellular staining using the BD Pharmingen Transcription Factor Buffer Set according to the manufacturer’s instructions. Following intracellular staining, cells were suspended in FACS buffer and analyzed using the CyAn ADP analyzer (Beckman Coulter). Data were analyzed using FlowJo to obtain the number of liver immune cells.

### Assessment of liver function.

Six hours after the liver I/R model, blood was collected from the apex of the heart. ALT (C009-2) and AST (C010-2) kits from Jiancheng Bioengineering Institute were used to measure the serum ALT and AST levels.

### Histological analysis.

After euthanasia, the ischemic liver lobes were fixed in 4% neutral-buffered formalin, embedded in paraffin, and sectioned into 5 μm slices. The slices were stained with H&E for histological analysis. The tissues were assessed for necrosis to characterize liver injury following I/R using Image-Pro Plus software (Media Cybernetics, Rockville, MD).

### Immunofluorescence.

Teffs and Tregs were isolated from the spleens of *Tlr4*^fl/fl^ mice and Treg-*Tlr4*^–/–^ mice using magnetic bead sorting. These cells were incubated overnight with an anti-TLR4 antibody and incubated with Cy5-conjugated secondary antibodies for 1 hour at room temperature. Cells were fixed in 4% paraformaldehyde and subsequently stained with 1 μg/mL DAPI for 10 minutes. Images were captured using a Leica confocal microscope (TCSSP8 STED3X, Germany) under consistent imaging settings for each antibody labeling experiment. Negative controls were used for gating.

### ELISA.

To determine IL-1β, TNF-α, and IL-10 levels, 100 μL of serum and 100 μL of the Antibody Cocktail were added to the corresponding ELISA kit wells (Abcam, Cat: ab197742, ab208348, and ab255729) and incubated for 1 hour at room temperature. After washing the wells 3 times, 100 μL of TMB substrate was added and left to incubate in the dark for 10 minutes. Subsequently, 100 μL of Stop Solution was added, followed by shaking the plate for 1 minute. Finally, the optical density (OD) at 450 nm was recorded.

### qPCR.

Total RNA was extracted from ischemia liver tissue and Tregs, then reverse-transcribed into cDNA using the RevertAid First Strand cDNA Synthesis Kit. qPCR was performed on a LightCycler 480 system (Roche, CA) using SYBR Green Master Mix, with β-actin as the internal control. Relative gene expression was calculated using the comparative threshold cycle (Ct) method (Relative gene expressio*n* = 2^−ΔΔCt^) (75). The specific gene primers used in this study are listed in Supplemental Table 3.

### Bulk RNA-seq.

The RNA of hepatic Tregs from WT mice before and following I/R, as well as from *Tlr4*^fl/fl^ mice and Treg-*Tlr4*^–/–^ mice following I/R, were extracted, enriched, and purified. RNA-seq was performed using the DNBSEQ platform. Hierarchical Indexing for Spliced Alignment of Transcripts (HISAT, v2.0.4) was used to map RNA-seq reads. Sequence count normalization, false discovery rate (FDR) adjustment, and differential gene expression (DEGs) analysis were performed. DEGs were defined with log_2_ (FC) > 1.5 and Q value < 0.05. DEGs analysis, gene ontology (GO) functional annotation analysis, Kyoto Encyclopedia of Genes and Genomes (KEGG) and were performed using the Dr. Tom network platform of BGI (http://report.bgi.com/) and the ShinyGO 0.77 network platform (http://bioinformatics.sdstate.edu/go/). Ingenuity pathway analysis (IPA, Ingenuity Systems, www.ingenuity.com) software was used for pathway analysis.

### Western Blot.

Total protein lysates were prepared from Tregs using a lysis buffer containing protease inhibitors. Protein concentrations were measured using the BCA method. Proteins were separated by SDS-PAGE and transferred to NC membranes. Blots were imaged at different exposure times. All quantitative data were derived from short-exposure images within the linear range to ensure accuracy, while a representative 5-minute exposure image is shown for FOXP3 to optimally visualize the protein bands. The primary antibodies used in this study are listed in the Supplemental material.

### Statistics.

All analyses were performed using Prism 8.0 (GraphPad Software, USA). Results are expressed as mean ± SEM. Comparisons were made using Student’s *t* test and ANOVA. Correlations were assessed using Spearman correlation analysis. The *P* < 0.05 was considered statistically significant.

### Study approval.

The protocol for this clinical study conformed to the ethical guidelines of the Declarations of Helsinki and Istanbul. All procedures and the use of liver tissue were approved by the Ethics Committee and Institutional Review Board of Wuhan Union Hospital, Huazhong University of Science and Technology (UHCT-IEC-SOP-016-03-01). All participants signed an informed consent form before participation in the study. Written informed consent was obtained from all participants. The experimental animal protocols complied with the Management Rules of the Chinese Ministry of Health and were approved by the Animal Care and Use Committee of Wuhan Union Hospital.

### Data availability.

All data generated or analyzed during this study are included in this published article and its supplemental materials. All data values supporting figures and analyses are included in the Supporting Data Values file. The RNA-seq data associated with this study has been deposited in NCBI Gene Expression Omnibus (PRJNA1206100 and PRJNA1206185) and are listed in Supplemental Table 4. The corresponding authors will provide any further details related to this paper upon request.

## Author contributions

Designing research studies: JZ, Hui Wang, and HZ. Conducting experiments: YZ, HZ, TR, CC, CT, and PS. Acquiring data: YZ, PX, GW, PS, CW, X Zhong, YG, Han Wang, X Zhang, and QZ. Analyzing data: YZ and TR. Writing-original draft preparation: YZ and CC. Writing-review and editing: YZ, HZ, JZ and Hui Wang. Supervision: JZ and Hui Wang. Funding acquisition: JZ, Hui Wang, YZ, and X Zhang. YZ and HZ are co-first authors and contributed equally to this work. All authors contributed to the article and approved the submitted version.

## Funding support

- The National Key Research and Development Program of China, Nos. 2024ZD0520805 (HW) and Nos.2024YFA1107601 (JZ).
- The National Natural Science Foundation of China (NSFC), nos. 82370647 (HW), 82170642 (JZ), 81801923 (X Zhang), 81670575 (JZ), 81570570 (HW), and 81070355 (JZ).
- The Program of HUST Academic Frontier Youth Team, Huazhong University of Science and Technology (2018QYTD02).
- The Open Foundation of Hubei Key Laboratory of Regenerative Medicine and Multi-disciplinary Translational Research (2022zsyx003).
- The Research Grant of Key Laboratory of Anesthesiology and Resuscitation (Huazhong University of Science and Technology), Ministry of Education [No.2024MZFS019]).

## Supplementary Material

Supplemental data

Unedited blot and gel images

Supporting data values

## Version 1 03/02/2026

Electronic publication
