# Supplementary material for: TLR4 maintains Treg-mediated protection against adverse outcomes in a model of hepatic surgical stress
Source: J Clin Invest. 2026 Mar 2;136(5):e194607. doi: 10.1172/JCI194607 (PMC12948431; doi:10.1172/JCI194607)
Supplement: Supplemental data [file jci-136-194607-s183.pdf]

Supplemental figures and tables

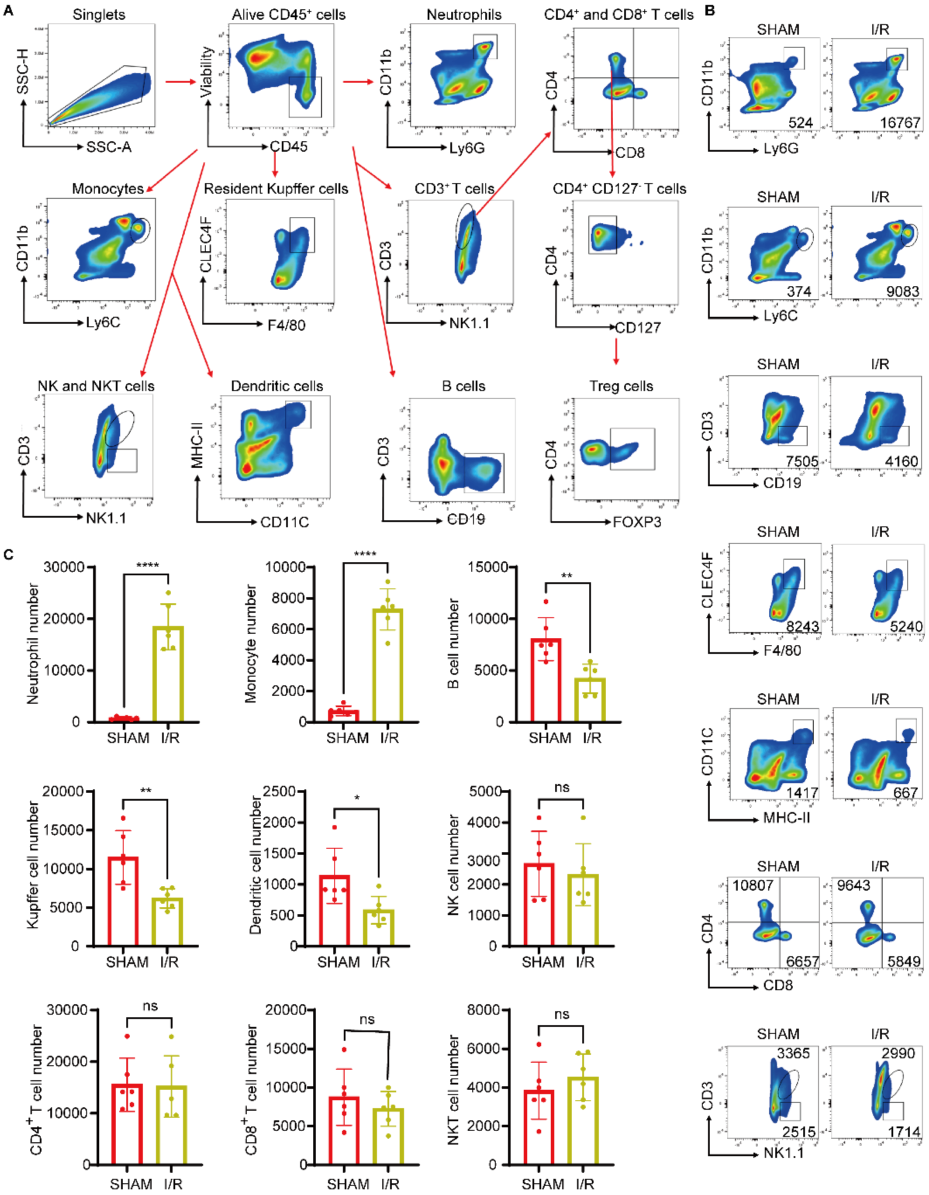

Supplemental Figure 1. Quantification of hepatic immune cells from sham and I/R WT mice. (**A**) Gating strategy for the identification of hepatic CD45^{+} immune cell populations by flow cytometry in sham and I/R WT mice. (**B-C**) Representative FACS plots and absolute counts of neutrophils, monocytes, dendritic cells, B cells, resident Kupffer cells, CD4^{+} T cells, CD8^{+} T cells, NK cells, and NKT cells per 1,000,000 hepatic non-parenchymal cells in sham and I/R WT mice (n=6 per group). Statistical analyses were performed using unpaired, two-tailed t-tests (**C**). *p < 0.05, **p < 0.01, ***p < 0.001; ***p < 0.0001; ns, no significant. I/R: ischemia/reperfusion.

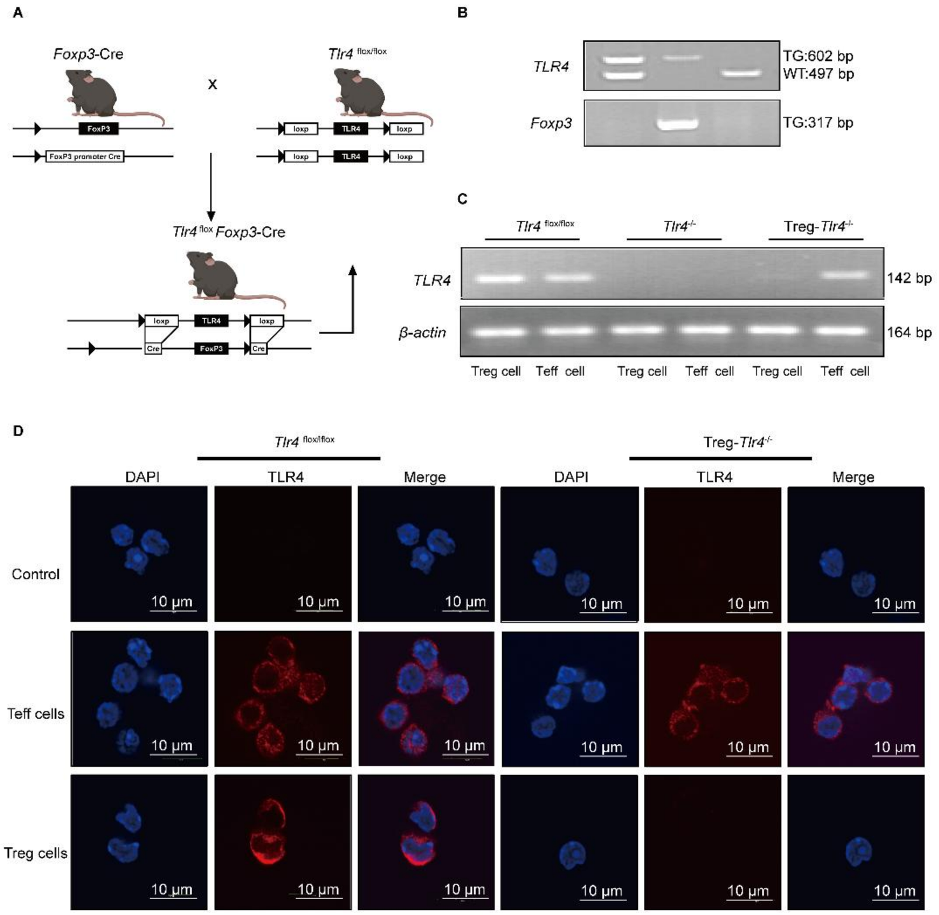

Supplemental Figure 2. Generation and identification of Treg-*Tlr4*$^{-/-}$ mice. (**A**) Schematic diagram illustrating the generation of Treg-*Tlr4*$^{-/-}$ mice. (**B**) PCR-based identification of DNA expression for *loxP*-flanked *TLR4* alleles and *Cre* recombinase expression to distinguish heterozygous *Tlr4*$^{flox/flox}$ mice, homozygous *Tlr4*$^{flox/flox}$ mice, and Treg-*Tlr4*$^{-/-}$ mice. (**C**) PCR-based analysis of *Tlr4* gene expression in Teffs and Tregs isolated from *Tlr4*$^{flox/flox}$ mice, *Tlr4*$^{-/-}$ mice, and Treg-*Tlr4*$^{-/-}$ mice. (**D**) Representative immunofluorescence images showing TLR4 expression (red) in Teffs and Tregs isolated from *Tlr4*$^{flox/flox}$ mice (left panel) and Treg-*Tlr4*$^{-/-}$ mice (right panel) through magnetic bead sorting. Scale bars, 10 μm. Tregs: regulatory T cells; Teffs: effector T cells.

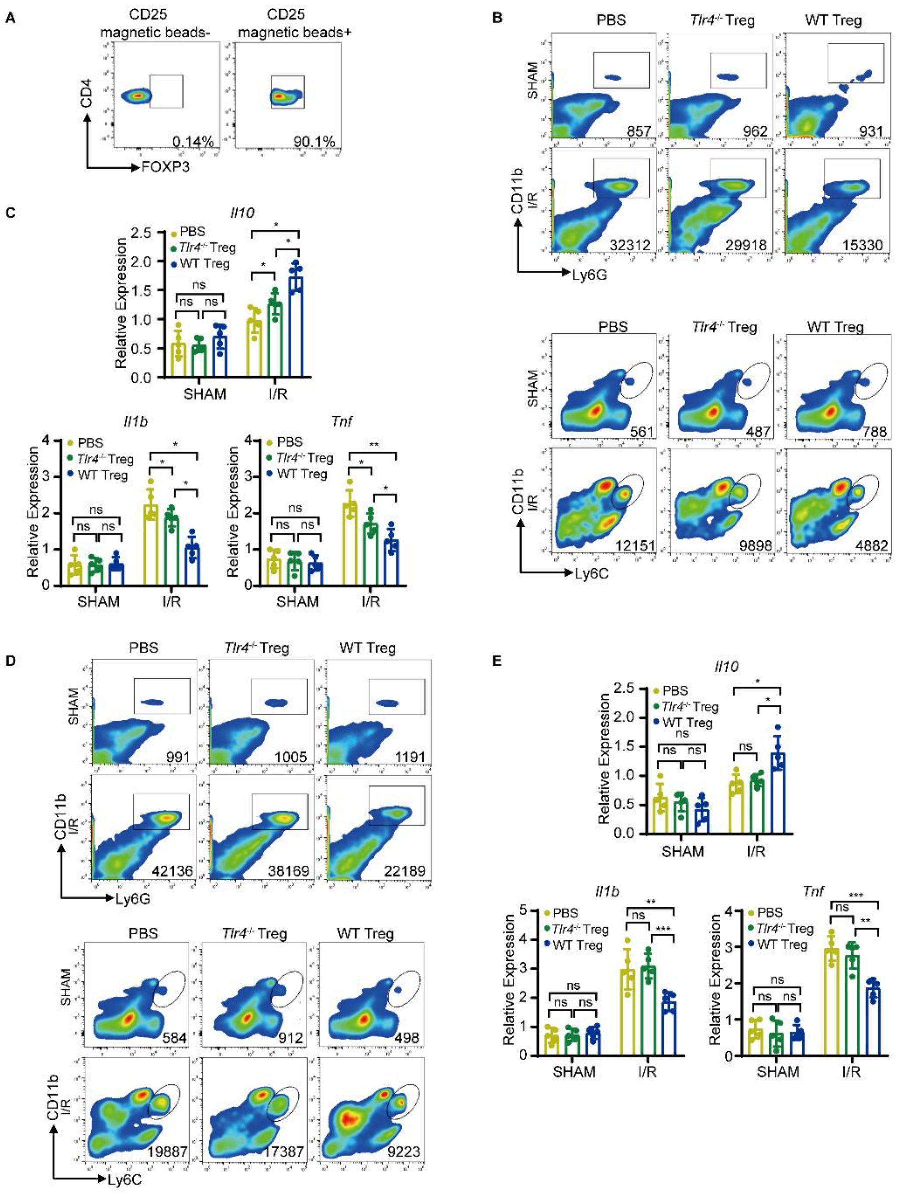

Supplemental Figure 3. *Tlr4* expression in Tregs alleviates liver I/R injury in Treg-depleted mice. (**A**) Representative flow cytometry plots showing the proportion of CD4$^+$FOXP3$^+$ Tregs in isolated Treg cells and control using MACS-sorting. (**B**) Representative flow cytometry plots of CD11b$^+$Ly6G$^+$ neutrophils and CD11b$^+$Ly6C$^+$ monocytes in Treg-depleted *Foxp3* DTR mice administered PBS, *Tlr4*$^{-/-}$ Tregs, or WT Tregs before and after liver I/R (n=5 per group). (**C**) Quantitative PCR analysis of *Il1b*, *Tnf*, and *Il10* mRNA expression in ischemic liver from Treg-depleted *Foxp3* DTR mice administered PBS, *Tlr4*$^{-/-}$ Tregs, or WT Tregs before and after liver I/R (n=5 per group). (**D**) Representative flow cytometry plots of CD11b$^+$Ly6G$^+$ neutrophils and CD11b$^+$Ly6C$^+$ monocytes in Treg-depleted WT mice through anti-CD25 antibody and then administered PBS, *Tlr4*$^{-/-}$ Tregs, or WT Tregs before and after liver I/R (n=5 per group). (**E**) Quantitative PCR analysis of *Il1b*, *Tnf*, and *Il10* mRNA expression in ischemic liver from Treg-depleted WT mice adoptively transferred with PBS, *Tlr4*$^{-/-}$ Tregs, or WT Tregs after I/R (n=5 per group). Statistical analyses were performed using one-way ANOVA with Tukey's post test (**C and E**). I/R: ischemia/reperfusion.

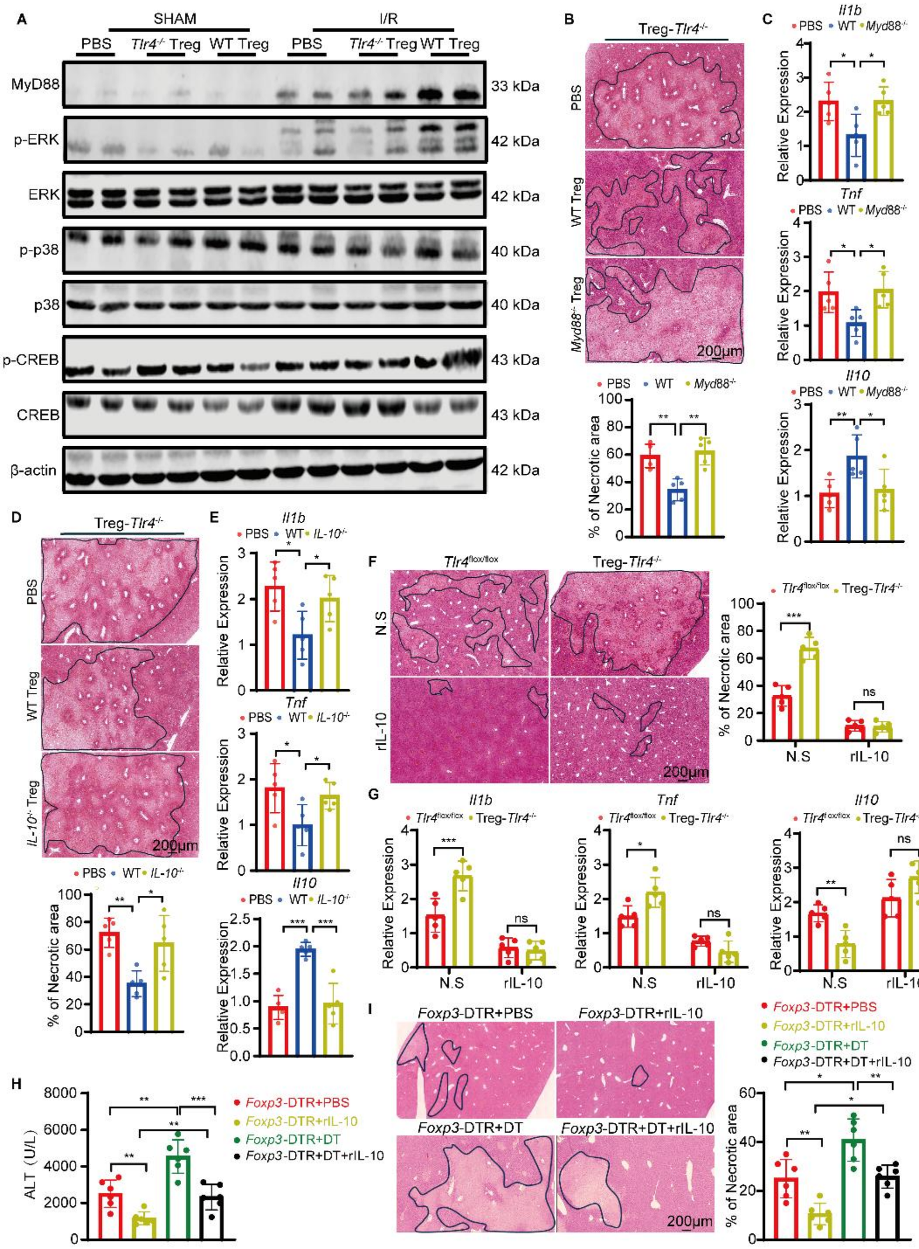

Supplemental Figure 4. TLR4/MyD88/ERK/CREB-mediated IL-10 production in Tregs alleviates liver I/R injury. (**A**) Western blot analysis of MyD88, p-ERK, p-p38, and p-CREB protein expression levels in Tregs from Treg-*Tlr4*$^{-/-}$ mice adoptively transferred with PBS, *Tlr4*$^{-/-}$ Tregs or WT Tregs before and after I/R. (**B**) Representative H&E staining images of ischemia liver from Treg-*Tlr4*$^{-/-}$ mice adoptively transferred with PBS, *Myd88*$^{-/-}$ Tregs or WT Tregs after liver I/R (n=5 per group). (**C**) Quantitative PCR analysis of *Il1b*, *Tnf*, and *Il10* mRNA expression in ischemia liver from Treg-*Tlr4*$^{-/-}$ mice adoptively transferred with PBS, *Myd88*$^{-/-}$ Tregs or WT Tregs after liver I/R (n=5 per group). (**D**) Representative H&E staining images of ischemia liver from Treg-*Tlr4*$^{-/-}$ mice adoptively transferred with PBS, *IL-10*$^{-/-}$ Tregs or WT Tregs after liver I/R (n=5 per group). (**E**) Quantitative PCR analysis of *Il1b*, *Tnf*, and *Il10* mRNA expression in ischemia liver from Treg-*Tlr4*$^{-/-}$ mice adoptively transferred with PBS, *IL-10*$^{-/-}$ Tregs or WT Tregs after liver I/R (n=5 per group). (**F**) Representative H&E staining images of ischemia liver lobe from *Tlr4*$^{flox/flox}$ mice and Treg-*Tlr4*$^{-/-}$ mice administered recombinant IL-10 or its control after I/R (n=5 per group). (**G**) Quantitative PCR analysis of *Il1b*, *Tnf*, and *Il10* mRNA expression in ischemia liver from *Tlr4*$^{flox/flox}$ mice and Treg-*Tlr4*$^{-/-}$ mice administered recombinant IL-10 or its control after I/R (n=5 per group). (**H**) Serum ALT levels in I/R *Foxp3* DTR mice following Treg depletion or not, and treatment with recombinant IL-10 or vehicle (n=6 per group). (**I**) Representative H&E staining images of ischemia liver from these mice (n=6 per group); Scale bars, 200 μm. Statistical analyses were performed using one-way ANOVA with Tukey's post test (**B-E**), two-way ANOVA with Sidak's post-test (**F-G**) and one-way ANOVA (**H-I**). *p < 0.05, **p < 0.01, ***p < 0.001; ns, no significant; ALT: Alanine aminotransferase; AST: Aspartate aminotransferase; I/R: ischemia/reperfusion; rIL-10: recombinant IL-10.

Supplemental Table 1. Preoperative and Postoperative clinical characteristics of patients with hepatic hemangioma (p values were determined using ANOVA test)

	**No hepatic portal block (N=6)**	**Hepatic portal block (<8.4min) (N=5)**	**Hepatic portal Block (>8.4min) (N=5)**	**p-value**
**Preoperative**				
Age – median (IQR)	49.5 (39-62)	48 (36-57)	50.2 (41-63)	0.92
Female gender – n (%)	3 (50%)	2 (40%)	3 (60%)	0.72
Hypertension– n (%)	0(0%)	0(0%)	1(20%)	0.46
Hyperlipidemia– n (%)	0(0%)	1(20%)	0 (0%)	0.46
Body mass index – median (IQR)	23.30 (19.92-30.67)	23.2 (19.31-24.8)	24.11 (21.65-25.39)	0.43
AST – median (IQR)	19.67 (15-27)	20 (17-22)	21(19-25)	0.89
ALT – median (IQR)	18.33 (9-28)	19.1 (8-25)	17.5 (11-28)	0.68
Total bilirubin – median (IQR)	11.38(5.3-18.8)	13.75 (7.2-19.6)	14.3 (9.2-20.5)	0.17
INR – median (IQR)	1.68(0.87-4.2)	1.70 (0.94-3.2)	1.74 (0.98-3.5)	0.94
Albumin – median (IQR)	40.35 (36.2-43.1)	41.78 (38.9-43.6)	44.1 (41.2-48.6)	0.17
platelet count– median (IQR)	178.5 (117-244) *10^9/L	179 (104-225) *10^9/L	176.4 (111-243) *10^9/L	0.93
PT–median (IQR)	12.8 (11.7-13.9)	11.85 (12.9-15.1)	11.85 (13-14.8)	0.57
APTT–median (IQR)	39.82 (29.9-42.9)	39.9 (33.1-40.8)	38.28 (32.5-41.4)	0.26
Hepatic pedicle block–median (IQR)	0(0) min	3.6 (1-8.4) min	10.2 (8.4-15) min	<0.01
**Postoperative**				

AST – median (IQR)	327.5 (91-655)	471.8 (268-694)	1015.6 (830-1284)	0.002
ALT – median (IQR)	323.67 (73-705)	597.2 (196-746)	1250 (933-1910)	0.005
Total bilirubin – median (IQR)	16.75(7.7-26)	18.36 (11-28.7)	21.7 (11.7-26)	0.47
INR – median (IQR)	1.58 (0.97-3.2)	1.77 (0.89-3.42)	1.84 (0.86-3.98)	0.06
Albumin – median (IQR)	34.45 (30.7-38)	37.26 (22.1-39.6)	35.71 (25.1-40.6)	0.85
platelet count– median (IQR)	191 (102-261) $*10^9/L$	183 (89-277) $*10^9/L$	195 (121-281) $*10^9/L$	0.71
PT–median (IQR)	13.1 (12.4-16.2)	16.5 (14.9-18.5)	19.5 (17.7-20.5)	0.04
APTT–median (IQR)	39.97 (29.9-41.9)	38.28 (32.5-38.4)	41.1 (38.5-43.4)	0.14

Supplemental Table 2. Antibodies

Name	Supplier	Cat no.	Clone no.
anti-CD45-FITC	BioLegend	103108	103108
anti-CD11b- Brilliant Violet 421™	BioLegend	101251	M1/70
anti-F4/80 Spark YG™ 593	BioLegend	101251	QA17A29
anti-CLEC4F Alexa Fluor® 647	BioLegend	156804	3E3F9
anti-CD19 BUV563	BD Biosciences	749028	1D3
anti-Ly6G- Alexa Fluor® 700	BioLegend	127622	1A8
anti-Ly6C- Brilliant Violet 510™	BioLegend	128033	HK1.4
anti-NK1.1- Brilliant Violet 570™	BioLegend	108733	PK136
anti-CD8a BUV496	BD Biosciences	750024	53-6.7
anti-CD4- BUV615	BD Biosciences	613006	GK1.5
anti-CD3-BUV661	BD Biosciences	741562	17A2
anti-CD11c Brilliant Violet 650™	BioLegend	117339	N418
anti-I-A/I-E Brilliant Violet 711™	BioLegend	107643	M5/114.15.2
anti-IL-10- Brilliant Violet 421™	BioLegend	505021	JES5-16E3
anti-CD127- PE-cy7	eBioscience™	25-1271-82	A7R34
anti-mouse FOXP3-PE	BioLegend	126404	MF-14
anti-mouse FOXP3-APC	eBioscience	17-5773-82	FJK-16s
anti-TLR1-BV510	BD Biosciences	743134	TR23
anti-TLR2- PE/Cyanine7	BioLegend	153012	QA16A01
anti-TLR3- Alexa Fluor® 700	Novus	NBP227405	27N3D4
anti-TLR4- BV786	BD Biosciences	741015	MTS510
anti-TLR5- Alexa Fluor® 647	BioLegend	148104	ACT5
anti-TLR6- BV650	BD Biosciences	743269	C1N2
anti-TLR7- PE/Cy5.5	Novus	N104349PEC5	4F4
anti-TLR8	Novus	DDX0481P100	307D3.01
anti-TLR9- PerCP	Novus	NB371746	26C593R
anti-TLR11-mFluor Violet 450	Novus	NB346482	786404
Anti-FOXP3 antibody	ABclonal	A5760	Polyclonal
anti-TLR4 antibody	eBioscience™	HTA125	14991782
Cy3-conjugated anti-mouse	Beyotime	Polyclonal	A0521
Cy5-conjugated anti-mouse	Sigma	Polyclonal	AC111S
anti-mouse CD25 antibody	Bio X cell	PC61	BE0012
anti-mouse CD16/32 Antibody	BioLegend	93	101302
Recombinant Mouse IL-10	R&D	417-ML-025/CF	/
Anti-MyD88 antibody	Abcam	ab2064	Polyclonal
Phospho-ERK1/2 antibody	Abclonal	AP0472	Polyclonal
ERK1/2 antibody	Abclonal	A16686	Polyclonal
Phospho-p38 MAPK-antibody	Abclonal	AP0526	Polyclonal
p38 MAPK-antibody	Abclonal	A5049	Polyclonal

Phosph- CREB antibody	Abclonal	AP0019	Polyclona
CREB antibody	Abclonal	A10826	Polyclonal
β-actin antibody	Cell Signaling Technology	4970	Polyclonal

Supplemental Table 3. Primers for genotyping and RT-qPCR (mouse)

**Name**	**Sequence**	**Supplier**
Tlr4$^{flox/flox}$ forward A	AGGGAGATGTGTGTGAAGAAG CC	Sangon Biotech
Tlr4$^{flox/flox}$ reverse B	TGACTTTCTCAGTTTGGTCCTGG	Sangon Biotech
Tlr4$^{flox/flox}$ reverse C	TTCCAGCTATGGCCCAGATGAACT	Sangon Biotech
Foxp3-Cre forward	CGGGTCAGAAAGAATGGTGT	Sangon Biotech
Foxp3-Cre reverse	CAGTTTCAGTCCCCATCCTC	Sangon Biotech
Tlr4 forward	CAAGAACATAGATCTGAGCTTCAA CCC	Sangon Biotech
Tlr4 reverse	GCTGTCCAATAGGGAAGCTTTCTAGAG	Sangon Biotech
Il1b forward	GGATGAGGACATGAGCACC	Sangon Biotech
Il1b reverse	GGAGCCTGTAGTGCAGTTGT	Sangon Biotech
Tnf forward	ACTGAACTTCGGGGTGATCG	Sangon Biotech
Tnf reverse	GGCTACAGGCTTGTCACTCG	Sangon Biotech
Il10 forward	CCCTTTGCTATGGTGTCCTT	Sangon Biotech
Il10 reverse	TGGTTTCTCTTCCCAAGACC	Sangon Biotech
Foxp3 forward	TGACCAAGGCTTCATCTGTG	Sangon Biotech
Foxp3 reverse	GAGGAACTCTGGGAATGTGC	Sangon Biotech
Ikzf2 forward	CAGCATGCACCAAGCACAAT	Sangon Biotech
Ikzf2 reverse	ATGGGGCCATCCATGTTGTT	Sangon Biotech

Il2ra forward	GCGTTGCTTAGGAAACTCCTGG	Sangon Biotech
Il2ra reverse	GCATAGACTGTGTTGGCTTCTGC	Sangon Biotech
Ctla4 forward	CTCAGCTGAACCTGGCTACC	Sangon Biotech
Ctla4 reverse	CCACTTGCAGACACCATTTG	Sangon Biotech
Myd88 forward	CATGTTCTCCATACCCTTGGT	Sangon Biotech
Myd88 reverse	AAACTGCGAGTGGGGTCAG	Sangon Biotech
Mapk1 forward	ACCCAAGTGATGAGCCCATTG	Sangon Biotech
Mapk1 reverse	TGCATTGAAAGTGCACACTGC	Sangon Biotech
Creb1 forward	GCTGCCTCTGGAGACGTACAA	Sangon Biotech
Creb1 reverse	GCTAGTGGGTGCTGTGCGA	Sangon Biotech
Mapk14 forward	GCATCGTGTGGCAGTTAAGA	Sangon Biotech
Mapk14 reverse	GTCCTTTTGGCGTGAATGAT	Sangon Biotech
β-actin forward	TGTTACCAACTGGGACGACA	Sangon Biotech
β-actin reverse	GGGGTGTTGAAGGTCTCAAA	Sangon Biotech

Supplemental Table 4. Deposited data

**Name of repository**	**Identifier**	**Link**
Bulk RNA-Seq for murine liver Treg cells before and after liver I/R	PRJNA1206100	https://www.ncbi.nlm.nih.gov/bioproject/PRJNA1206100/
Bulk RNA-Seq for murine liver Treg cells in *Tlr4*$^{flox/flox}$ and Treg-*Tlr4*$^{-/-}$ after liver I/R	PRJNA1206185	https://www.ncbi.nlm.nih.gov/bioproject/PRJNA1206185/
