# Supplementary figures and images for: TLR4 maintains Treg-mediated protection against adverse outcomes in a model of hepatic surgical stress
Source: J Clin Invest. 2026 Mar 2;136(5):e194607. doi: 10.1172/JCI194607 (PMC12948431; doi:10.1172/JCI194607)

Full unedited blot/gel for Figure 1C

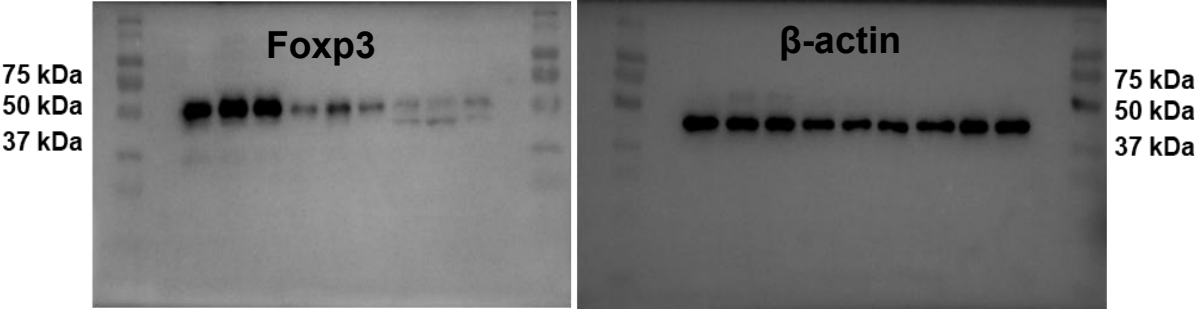

Full unedited blot/gel for Figure 7C

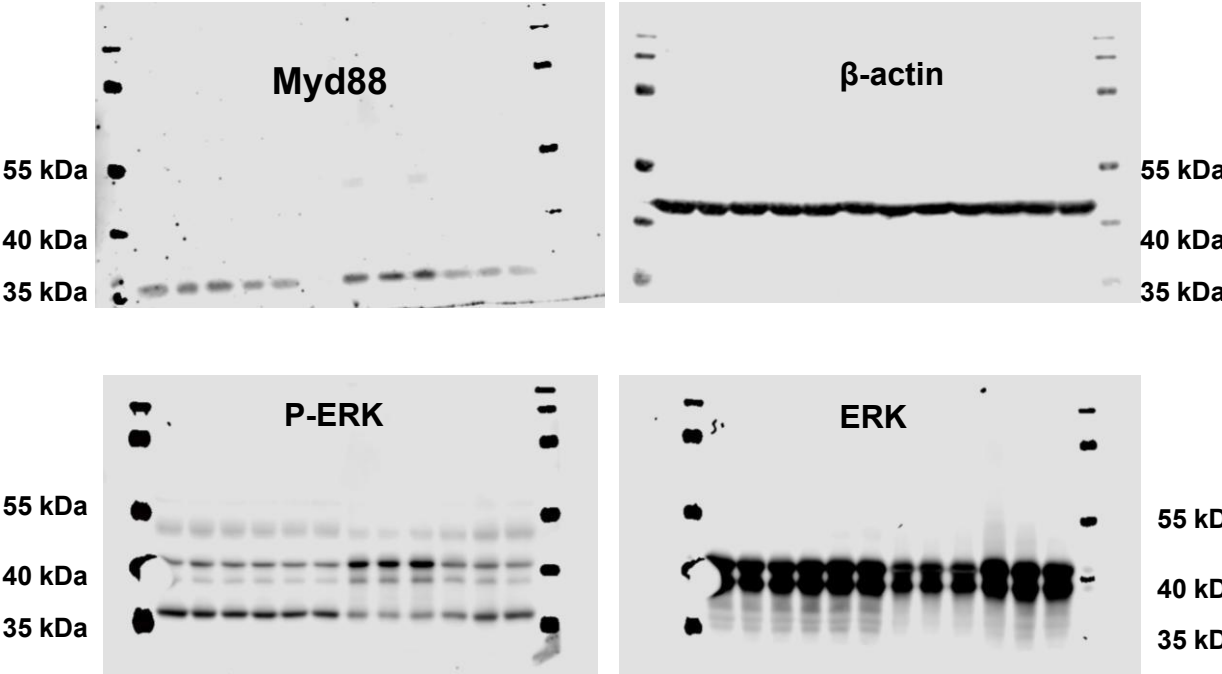

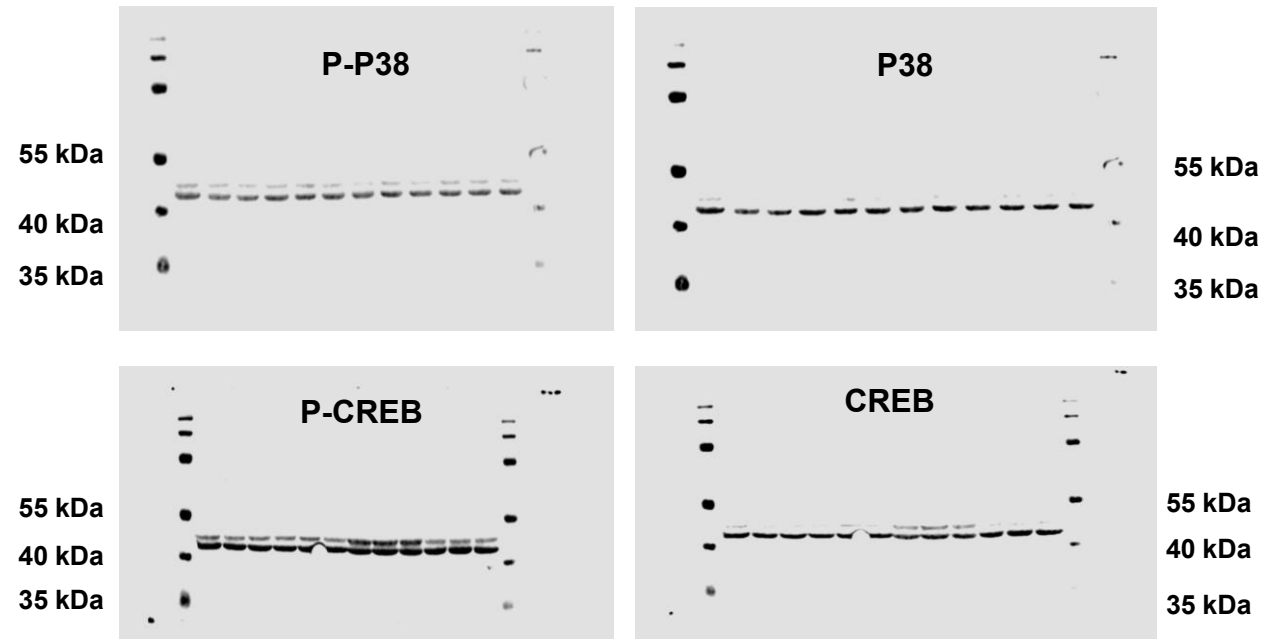

Supplement: Unedited blot and gel images [file jci-136-194607-s184.pdf]
